# Application of Bis(amido)alkyl Magnesiates toward the Synthesis of Molecular Rubidium and Cesium Hydrido-magnesiates

**DOI:** 10.1021/acs.organomet.4c00190

**Published:** 2024-06-13

**Authors:** Thomas X. Gentner, Gerd M. Ballmann, Sumanta Banerjee, Alan R. Kennedy, Stuart D. Robertson, Robert E. Mulvey

**Affiliations:** WestCHEM, Department of Pure and Applied Chemistry, University of Strathclyde, Glasgow G1 1XL, U.K.

## Abstract

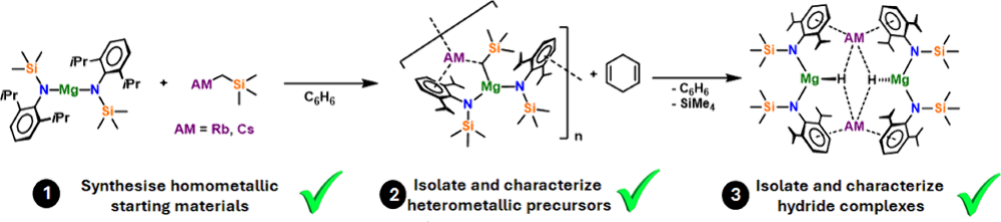

Rubidium and cesium are the least studied naturally occurring s-block metals in organometallic chemistry but are in plentiful supply from a sustainability viewpoint as highlighted in the periodic table of natural elements published by the European Chemical Society. This underdevelopment reflects the phenomenal success of organometallic compounds of lithium, sodium, and potassium, but interest in heavier congeners has started to grow. Here, the synthesis and structures of rubidium and cesium bis(amido)alkyl magnesiates [(AM)MgN′_2_alkyl]_∞_, where N′ is the simple heteroamide ^–^N(SiMe_3_)(Dipp), and alkyl is *n*Bu or CH_2_SiMe_3_, are reported. More stable than their *n*Bu analogues, the reactivities of the CH_2_SiMe_3_ magnesiates toward 1,4-cyclohexadiene are revealed. Though both reactions produce target hydrido-magnesiates [(AM)MgN′_2_H]_2_ in crystalline form amenable to X-ray diffraction study, the cesium compound could only be formed in a trace quantity. These studies showed that the bulk of the ^–^N(SiMe_3_)(Dipp) ligand was sufficient to restrict both compounds to dimeric structures. Bearing some resemblance to inverse crown complexes, each structure has [(AM)(N)(Mg)(N)]_2_ ring cores but differ in having no AM-N bonds, instead Rb and Cs complete the rings by engaging in multihapto interactions with Dipp π-clouds. Moreover, their hydride ions occupy μ_3_-(AM)_2_Mg environments, compared to μ_2_-Mg_2_ environments in inverse crowns.

## Introduction

Though not often explicitly expressed as such, alkali metal mediation (AMM), for example, a reaction mediated by the presence of an alkali metal intermediate has long been a common strategy employed in synthetic campaigns.^1^ Lithium, the lightest alkali metal, has been at the forefront of AMM, though both sodium and potassium have also made significant contributions to AMM down the years. Recently, an emerging focus on molecular main group hydrides,^2^ and s-block hydrides in particular, has broadened the scope of AMM to include the much rarer studied heavier alkali metals rubidium and cesium.^3^ Moreover, these heavier alkali metals are also finding application in unlikely AMM reactions such as the reduction of lithium cations to lithium metal.^4^ Furthermore, the European Chemical Society’s most recent version of the periodic table of elemental scarcity confirms the availability of Rb and Cs as “plentiful supply”.^5^ Therefore, heavyweight AM chemistry is likely to intensify in the coming years. Recent noteworthy results have been reported in homogeneous catalytic AMM applications of imines to amines and alkenes to alkanes where heavier alkali metal reagents are found to be the most effective alkali metal precatalysts.^6^ The latter study was inspired by Harder’s report that heavy Group 2 Ae(HMDS)_2_ complexes [Ae = Ca, Sr, Ba; HMDS = 1,1,1,3,3,3-hexamethyldisilazide, ^–^N(SiMe_3_)_2_] successfully accomplished alkene to alkane transformations via transfer hydrogenation, though significantly the lighter Mg(HMDS)_2_ failed under similar conditions.^7^ Subsequently, we found that AMM repaired this Mg(HMDS)_2_ inertness via the magnesiates RbMg(HMDS)_3_ and CsMg(HMDS)_3_, both of which outperformed their lighter congeners in a catalytic cycle involving heteroleptic Rb/Cs(HMDS)_2_(H) hydride intermediates.^6a^

More fundamental development of rubidium and cesium amide chemistry is clearly required from the promise of these preliminary findings. Our previous work reported the isolation of the benzene solvate [{(C_6_H_6_)RbMg(HMDS)_2_H}_2_]_∞_, the first example of a well-defined organorubidium hydride, which exists as an infinite chain structure in the solid state.^6a^ This new study posed the question, “by modifying the steric and electronic structure of the amide, for example, by incorporation of a π-surface within it, especially appealing to soft alkali metals, could we design discrete molecular heteroleptic amide-hydride Rb and Cs magnesiate species free of any solvating solvent ligands and where the amide is of a simple monodentate type?”

## Results and Discussion

### Synthesis of Bimetallic Infinite Supramolecular Structures

**Scheme 1:**
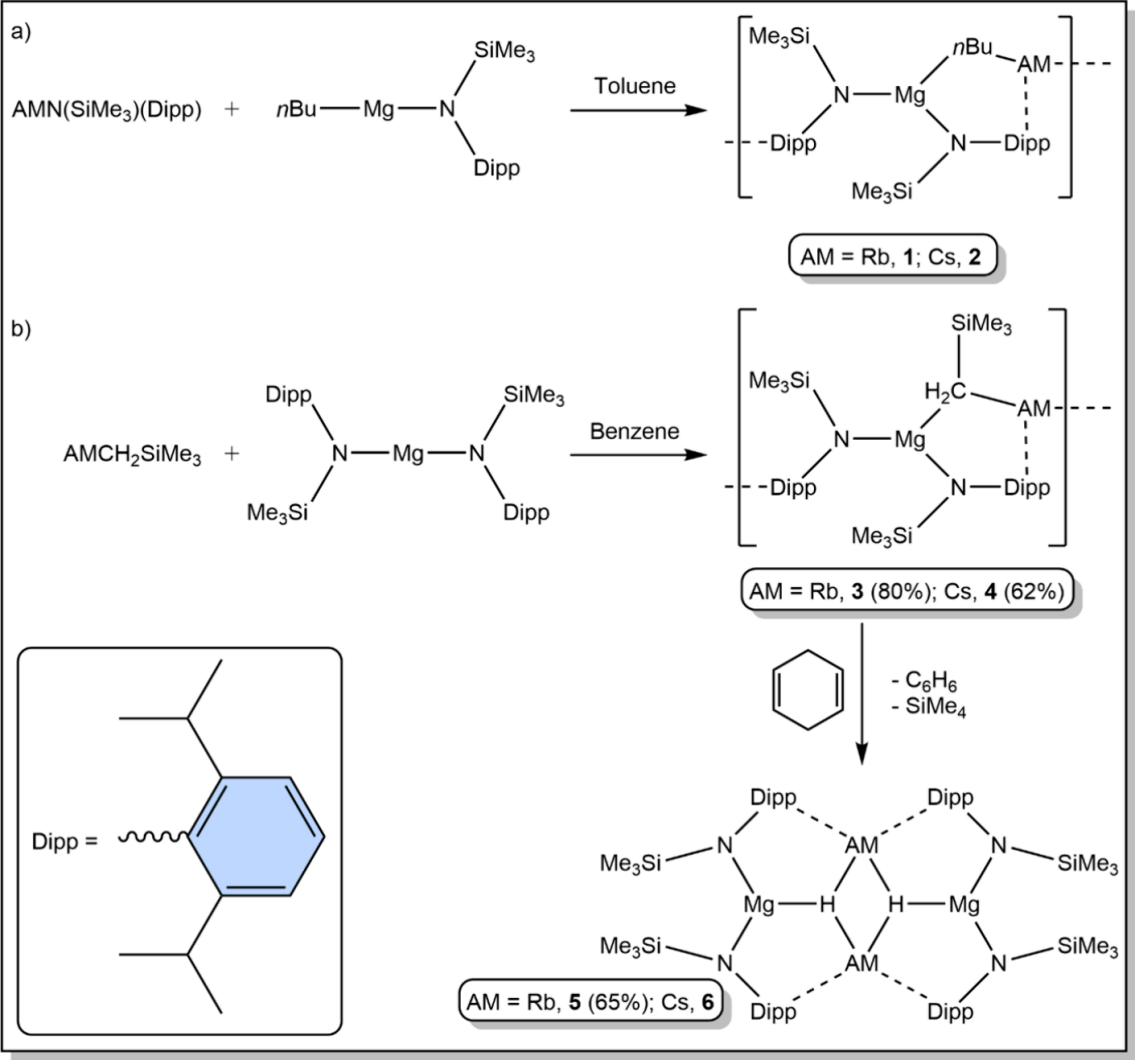
Cocomplexation Syntheses of Alkali Metal Bis(amido)alkyl Magnesiates **1**–**4** and Conversion of **3** and **4** into Bis(amido)hydrido-magnesiate Dimers **5** and **6** with Yields Shown where Pure Tangible Product Could be Obtained

**Figure 1:**
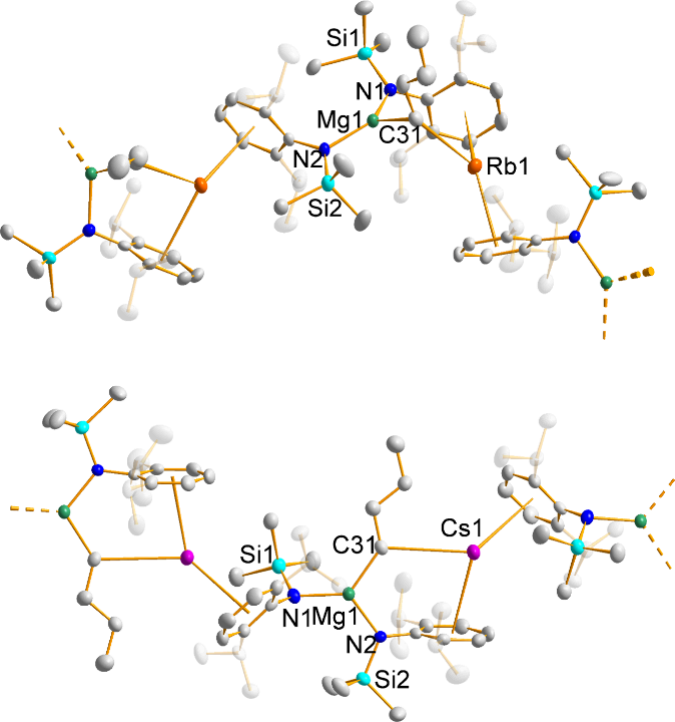
Sections of the infinite supramolecular chains of [(AM)MgN′_2_*n*Bu]_∞_ (AM = Rb, **1**, top; Cs, **2**, bottom) with thermal ellipsoids drawn at 50% probability and all hydrogen atoms omitted for clarity.

**Figure 2:**
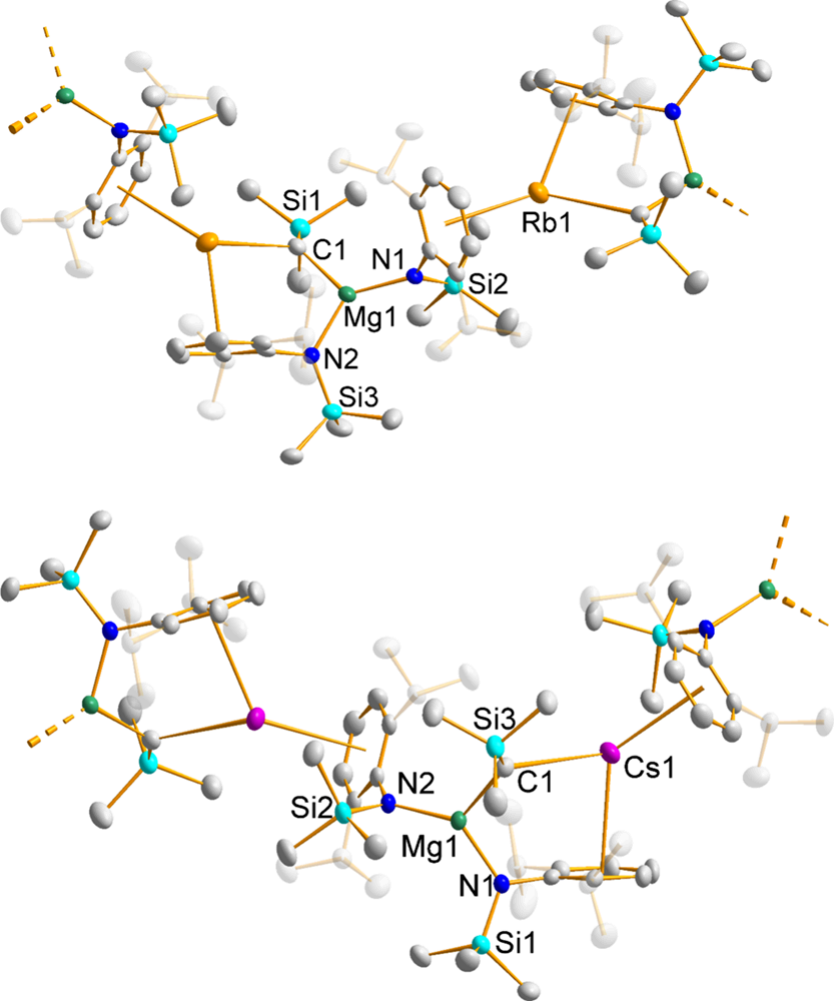
Sections of the infinite supramolecular chains of [(AM)MgN′_2_CH_2_SiMe_3_]_∞_ (AM = Rb, **3**, top; Cs, **4**, bottom) with thermal ellipsoids drawn at 50% probability and all hydrogen atoms omitted for clarity.

For this study, we decided to utilize the secondary heteroamido ligand ^–^N(SiMe_3_)(Dipp) (which we will refer to as N′; Dipp = 2,6-diisopropylphenyl), that is, replacing one of the SiMe_3_ groups of HMDS by an aromatic group with its π-surface. The alkali metal chemistry of this ligand is quite well-established,^8^ and crucially to this pursuit, the infinite supramolecular structure of unsolvated CsN(SiMe_3_)(Dipp)^9^ propagates in the solid state via multihapto Cs–C_6_(π) interactions, while its anionic nitrogen is naked in the sense that it does not form a bond to cesium, suggesting that it is potentially well-suited to our needs described above. Furthermore, the Dipp entity is a common feature of a wide variety of subvalent aluminum dimers which appear stitched together by heavy alkali metal cations engaging intramolecularly with *N*-bound Dipp groups at the periphery of the discrete molecules.^10^ Such interactions tend to be favored over similar interactions in reactions with external aromatic solvent molecules (for example, benzene, toluene, and so forth), which allow these organometallic species to propagate into extended supramolecular structures, thus enhancing the opportunities of us accessing discrete molecules. We, thus, targeted heteroleptic alkyl/bis-amido alkali metal magnesiates [(AM)MgN′_2_R; AM = Rb, Cs; R = alkyl group] as potential precursors to our desired amide-hydride complexes via a ligand exchange process. A cocomplexation reaction of *n*BuMgN′^11^ and (AM)N′ (Scheme 1a), a route successfully employed previously to prepare the sodium and potassium derivatives,^8d^ was run in toluene. Only a small crop of crystals resulted in each case, limiting our initial study to XRD and ^1^H NMR spectroscopy. ^1^H NMR spectroscopic analysis of the resulting products **1** and **2** in C_6_D_6_ confirmed the empirical formula of each complex. Particularly useful in this regard were the unobstructed resonances of the *i*Pr CH and AM-CH_2_ groups at approximately 4.0 and −1.5 ppm, respectively, which integrated to the expected 4:2 ratio commensurate with a 2:1 ratio of amido to alkyl ligands. X-ray diffraction (XRD) studies on single crystals of the two complexes confirmed them to be extended supramolecular structures of formula [(AM)MgN′_2_*n*Bu]_∞_ (see Figure 1 and crystallographic discussion section for full details). However, as already mentioned, the yields were poor, probably a reflection of the decreasing stability of *n*-butyl compounds as group one is descended with *n*-butyllithium being a reactive iconic organometallic reagent^12^ to *n*-butylcesium being, to the best of our knowledge, as yet unknown. Therefore, we decided to pursue an alternative less-reactive alkyl group in the hope of isolating a higher yielding heteroleptic magnesiate for onward reactivity studies, pivoting to trimethylsilylmethyl (Me_3_SiCH_2−_) as the alkyl anion.^13^ Because the alkali metal complexes of this anionic ligand are accessible, we could commence with the homoleptic MgN′_2_ as starting material, again exploiting a cocomplexation approach (Scheme 1b). Stirring a mixture of the two reagents in hexane yielded a precipitate which could be collected by filtration and recrystallized from hot benzene to yield the product [(AM)MgN′_2_CH_2_SiMe_3_]_∞_ (AM = Rb, **3**; Cs, **4**). ^1^H NMR spectroscopic analysis in C_6_D_6_ with 20% THF added to aid solubility confirmed the heteroleptic nature of the products. Again, the unobstructed resonances of the *i*Pr CH and AM-CH_2_ groups at approximately 4.0 and −1.5 ppm, respectively, were informative, confirming the 2:1 ratio of amido to alkyl ligands (see SI for full details of NMR spectroscopic analysis). The benzene recrystallization yielded single crystals in good yield (80 and 62%, respectively) suitable for XRD experiments which determined the supramolecular natures of both complexes **3** and **4** (Figure 2).

### X-ray Diffraction Studies on Complexes **1**–**4**

Bis-amido alkyl alkali metal magnesium complexes **1**–**4** share a common infinite supramolecular structural architecture, where the asymmetric unit contains a distorted trigonal-planar magnesium surrounded by three anionic ligands via σ-bonds to N (x 2) and C, while the alkali metal is also three-coordinate, but its σ-bond to the CH_2_^–^ carbanion is supplemented by interactions with the π-cloud of the aromatic Dipp rings of two amides (belonging to N1 and N2′, thus propagating the asymmetric unit along the polymeric chain). These AM–Dipp interactions can be considered of η^6^ hapticity, with the two Rb complexes having an average AM–C_6centroid_ distance of 3.036 Å and the AM–C distances in the range 3.167(4)–3.541(4) Å, while for the larger Cs cation, the corresponding values are as expected longer at 3.221 Å and 3.400(4)–3.772(4) Å. There is little variation in the AM–Dipp interactions as a function of the alkyl group, as is discernible from the data compiled in Table 1.

**Table 1:** Selected Bond Metrics (Å and °) of Rubidium and Cesium Bis(amido)alkyl Magnesiates **1**–**4**

	1 (AM = Rb)	2 (AM = Cs)	3 (AM = Rb)	4 (AM = Cs)
AM1–C_alkyl_	3.169(4)	3.516(7)	3.338(4)	3.441(3)
AM1–Ar_N1_	3.023(1)	3.232(1)	3.050(1)	3.201(1)
AM1–Ar_N2_	3.027(1)	3.232(1)	3.045(1)	3.187(3)
Mg1–C_alkyl_	2.149(4)	2.143(7)	2.158(4)	2.151(3)
Mg1–N1	2.023(3)	2.070(5)	2.044(3)	2.037(3)
Mg1–N2	2.040(3)	2.024(5)	2.034(4)	2.059(2)
C_alkyl_–AM1–Ar_N1_	89.4(1)	134.1(1)	120.2(1)	84.5(1)
C_alkyl_–AM1–Ar_N2_	121.6(1)	80.0(0)	86.8(1)	119.8(1)
Ar–AM1–Ar	145.5(1)	133.5(1)	129.6(1)	128.8(1)
C_alkyl_–Mg1–N1	109.4(1)	127.2(3)	124.7(1)	107.0(1)
C_alkyl_–Mg1–N2	115.8(1)	108.6(3)	106.5(2)	123.8(1)
N1–Mg1–N2	134.7(1)	123.8(2)	128.5(1)	128.7(1)

### Application of Magnesiates toward the Synthesis of Molecular Hydrides

**Figure 3:**
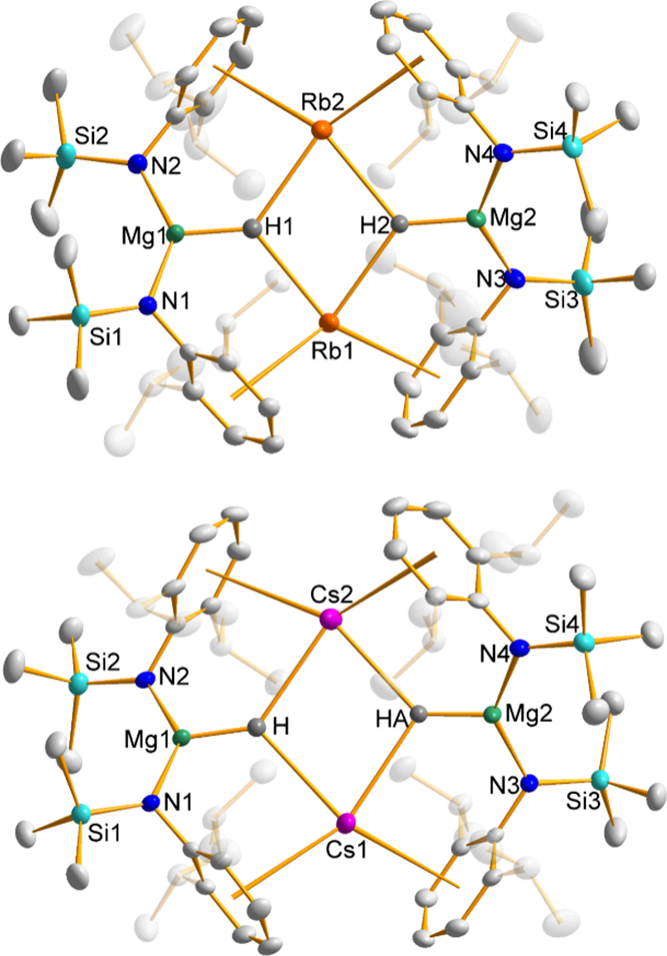
Molecular structures of hydrido-magnesiates [(AM)MgN′_2_H]_2_ (AM = Rb, **5**, top; Cs, **6**, bottom) with thermal ellipsoids drawn at 50% probability and all hydrogen atoms other than metal-bound hydrides omitted for clarity.

With high-yielding access to Rb and Cs bis-amido alkyl magnesiates in the form of **3** and **4** to hand, we turned our attention to their possible conversion into bis-amido hydrido-magnesiates. Gratifyingly, the reaction of **3** with a 4-fold excess of 1,4-cyclohexadiene in benzene solution at room temperature after filtration of intractable solids afforded a white powder (**5**), recrystallization of which followed by XRD analysis revealed the product to be the target compound [RbMgN′_2_H]_2_ (Figure 3, see Table 2 for selected bond parameters). Specifically, the alkyl ligand has been replaced with a hydrido ligand as intended, a substitution that leads to a change from an infinite supramolecular structure to a discrete dimeric structure. Though an identical procedure to that used to prepare hydrido-magnesiate **5** was followed using the cesium congener [CsMgN′_2_CH_2_SiMe_3_]_∞_ (**4**), no appreciable amount of pure solid could be obtained from the reaction solution. Many modifications of this reaction were attempted, for example, variation of reaction time, temperature, solvent, and hydride source, but none of these changes provided access to a tangible product with one exception. In that case, we were able to isolate a small number of single crystals which were shown by single-crystal XRD to be the target Cs hydrido-magnesiate (**6**, Figure 3). Notwithstanding, this success proved unrepeatable, and so no tangible amount of this product amenable to NMR spectroscopic interrogation was possible.

**Table 2:** Selected Bond Metrics (Å and °) of Rubidium and Cesium Hydrido-magnesiates **5** and **6**

	5 (AM = Rb)	6 (AM = Cs)		5 (AM = Rb)	6 (AM = Cs)
Mg1–H	1.76(2)	1.71(4)	Mg2–H_A_	1.75(2)	1.68(4)
Mg1–N1	2.005(1)	2.007(3)	Mg2–N3	1.999(1)	2.009(3)
Mg1–N2	2.000(1)	2.006(3)	Mg2–N4	2.001(1)	2.003(3)
AM1–Ar_N1_	3.070(1)	3.188(6)	AM2–Ar_N2_	3.053(1)	3.277(5)
AM1–Ar_N3_	3.053(1)	3.211(5)	AM2–Ar_N4_	3.099(1)	3.300(5)
AM1–H	2.77(2)	3.01(4)	AM2–H	2.75(2)	2.97(4)
AM1–H_A_	2.77(2)	2.98(4)	AM2–H_A_	2.77(2)	2.93(4)
N1–Mg1–N2	136.4(1)	135.2(1)	N3–Mg2–N4	136.0(1)	137.0(1)
N1–Mg1–H	113.5(7)	114.4(13)	N3–Mg2–H_A_	111.9(6)	111.1(13)
N2–Mg1–H	110.1(7)	110.4(12)	N4–Mg2–H_A_	112.1(6)	111.9(12)
AM1–H–Mg1	125.4(11)	125.1(18)	AM1–H_A_–Mg2	129.4(9)	121.9(17)
AM2–H–Mg1	129.0(11)	132.4(18)	AM2–H_A_–Mg2	125.6(9)	133.5(18)
AM1–H–AM2	105.6(7)	102.4(11)	AM1–H_A_–AM2	104.7(6)	104.0(11)
H–AM1–H_A_	74.5(6)	75.9(10)	H–AM2–H_A_	74.7(6)	77.3(10)
H–AM1–Ar_N1_	87.3(4)	83.0(7)	H–AM2–Ar_N2_	85.7(5)	78.4(7)
H–AM1–Ar_N3_	149.2(5)	142.6(7)	H–AM2–Ar_N4_	150.0(5)	140.6(7)
H_A_–AM1–Ar_N1_	147.2(4)	155.4(7)	H_A_–AM2–Ar_N2_	153.0(4)	149.8(7)
H_A_–AM1–Ar_N3_	83.5(4)	83.9(7)	H_A_–AM2–Ar_N4_	85.9(4)	78.0(7)
Ar_N1_–AM1–Ar_N3_	121.8(1)	120.8(1)	Ar_N2_–AM2–Ar_N4_	118.9(1)	131.9(2)
Mg1–H–AM1	125.4(11)	125.1(18)	Mg2–H_A_–AM1	129.4(9)	121.9(17)
Mg1–H–AM2	129.0(11)	132.4(18)	Mg2–H_A_–AM2	125.6(9)	133.5(18)
AM1-H–AM2	105.6(7)	102.4(11)	AM1–H_A_–AM2	104.7(6)	104.0(11)

**Figure 4:**
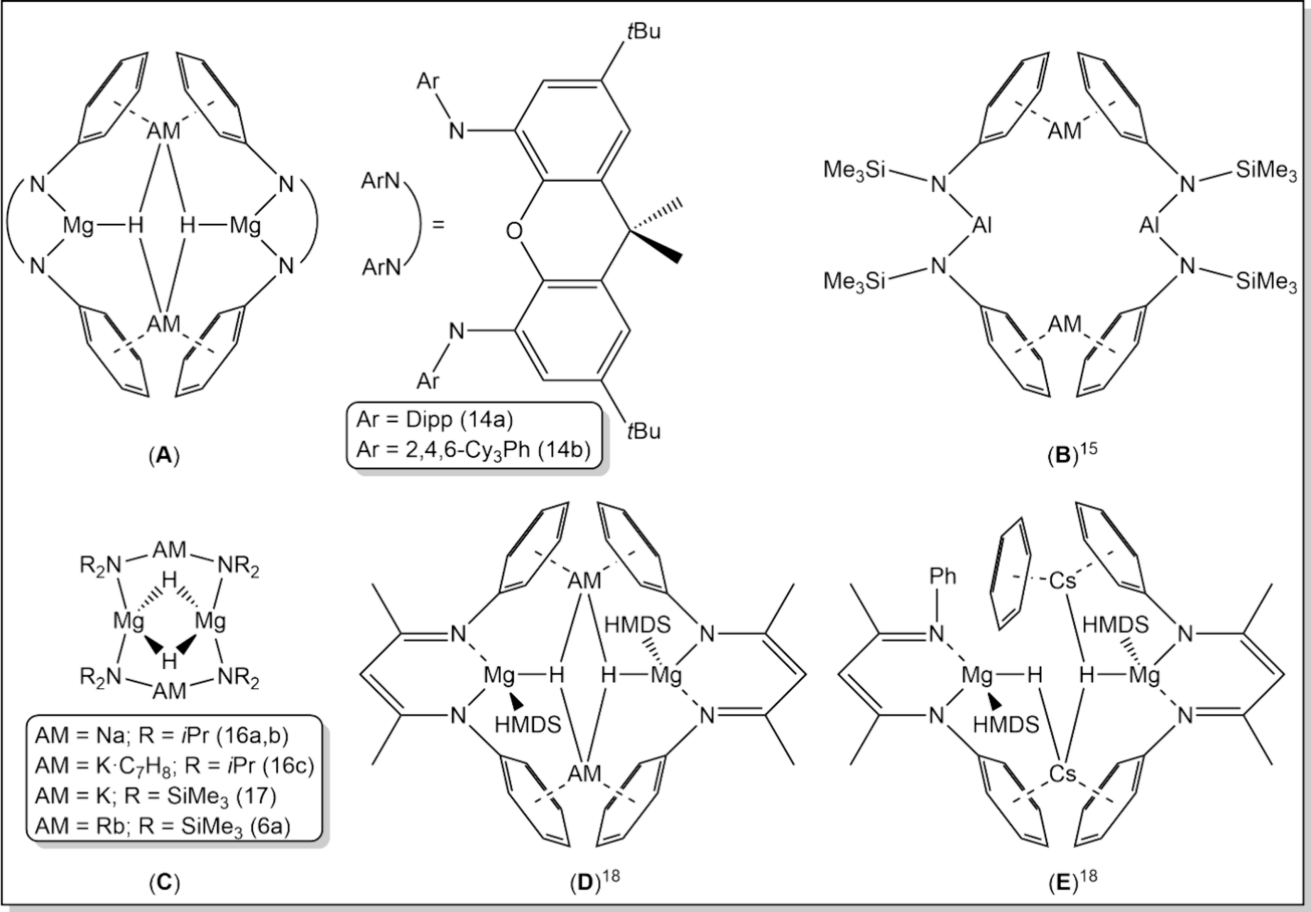
Crystallographically characterized alkali metal hydride complexes related to **5** and **6**. *N*-bound Dipp groups have been simplified as phenyl groups for clarity.

Hydrido-magnesiates **5** and **6** form crystallograpically noncentrosymmetric dimers in the solid state consisting of a bis-amido magnesium at each end, encompassing a rhomboidal (AM)_2_H_2_ unit in the center. The hydride is bound to the Mg center giving the planar group 2 metal center a coordination number of three (Σ< = 360° in all cases), while alkali metal cations lie sandwiched between the neutral cisoid-positioned aromatic Dipp rings of the magnesium-bound amides with long-range AM-H interactions, giving a dimeric alkali metal magnesiate formulation. This motif has been seen previously for K and Rb complexes utilizing a bidentate bis-amido dianion as the supporting ligand (**A**, Figure 4)^14^ but not previously with acyclic monoanionic amides. Heavier alkali metals frequently bridge two aromatic rings in a plethora of recently reported subvalent alkali metal Al(I) structures, although such complexes have no hydride ligand for the sake of charge balance.^10g−10i^ Again, these are dominated by supporting bidentate bis-amido dianions, with the first acyclic example reported by Hicks and Liptrot as recently as 2023 who, like us, have also exploited the ^–^N(SiMe_3_)(Dipp) amide (**B**).^15^

**Figure 5:**
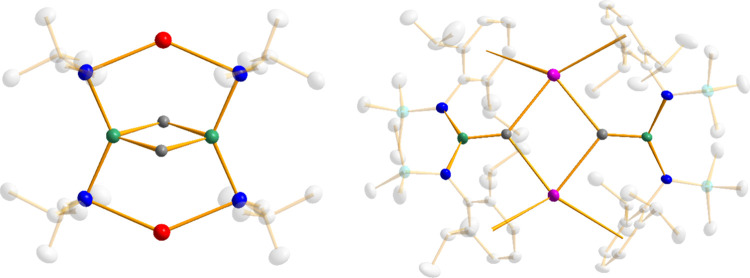
Contrast of [(AM)(N)(Mg)(N)]_2_ units encapsulating two hydride ligands where AM = Na and amide is aliphatic (left) and AM = Cs and amide is aromatic (right).

Interestingly, an analogy can be drawn between the structures of **5** and **6** with those belonging to the class of compounds known as “inverse crowns”, specifically to hydrido examples (**C**). The first hydrido inverse crowns of formula [(AM)_2_Mg_2_(NR_2_)_4_(H)_2_] (AM = Na, K; R = *i*Pr) were prepared by our group,^16^ while [(AM)_2_Mg_2_(HMDS)_4_(H)_2_] was reported later for K by the Hill group^17^ and for Rb by us.^6a^ It should be noted that the synthetic methods used to access these compounds were different, by β-H elimination from one N-*i*Pr unit of the tris-amide (AM)Mg(N*i*Pr_2_)_3_ for the diisopropylamides and by Mg–C/Si–H σ-bond metathesis between (AM)Mg(HMDS)_2_*n*Bu and PhSiH_3_ for the HMDS amides, and both these methods differ from the cyclohexadiene-deprotonation/hydride-transfer approach used to access **5** and **6**. All three of these inverse crown compounds have 8-atom [(AM)(N)(Mg)(N)]_2_ ring cores with two hydride ions each forming a bridge between two magnesium centers. These are closed rings in the sense that all their four metal atoms form bonds directly to the amido nitrogen atoms (Figure 5). Each of the 8-atoms in [(AM)(N)(Mg)(N)]_2_ and the two encapsulated hydrides are also present in structures **5** and **6**, but the rings have a discontinuity since the rubidium and cesium atoms do not bond directly to nitrogen atoms but bridge to these amide anions through their Dipp side arm. This reflects the penchant of these soft polarizable metals for engaging in multihapto interactions with π-electron clouds. A second feature distinguishing structures **5** and **6** from the inverse crowns is that their hydrides do not bridge between the two Mg centers but instead each forms a bridge between one Mg center and two AM centers, reflecting the larger sizes of Rb, Cs (compared to Li, Na, and K), and the N(SiMe_3_)(Dipp) amide [compared to N(SiMe_3_)_2_] which push the magnesiate anions further apart from each other.

Related to complexes **5** and **6**, Jones and co-workers recently reported a family of dinuclear heavy alkali metal magnesium hydrides supported by the bidentate ^Dipp^NacNac anion and the HMDS anion for a coordination number of four at magnesium (**D**). Interestingly, the Cs complex is more asymmetric than its lighter congeners since one of the Dipp side arms of the NacNac ligand is disengaged from one of the Cs atoms, being replaced instead by a molecule of aromatic benzene (**E**).^18^

In **5** and **6**, the hydride ligands can formally be considered as bonding primarily to Mg to give an anionic magnesiate center, as evidenced by their relatively short Mg–H bond lengths (mean: 1.755/1.695 Å, respectively). The AM–H distances are considerably longer at 2.765 and 2.973 Å for **5** and **6**, respectively. These Mg–H values are marginally shorter than those seen in Jones’ complexes **D** and **E** which display distances in the range 1.86(3)–1.91(2) Å, reflecting the lower coordination number (3) and altered electronics in our non-donor-chelated complexes versus such 4-coordinate, chelating complexes. The AM–H distances in **5** and **6** are comparable with those of Jones [2.79–2.87 Å (Rb) and 2.97–3.04 Å (Cs)]. The alkali metal aryl interactions are best described as η^6^ with the Rb–C distances in the range 3.233(2)–3.527(2) Å and Cs–C distances in the range 3.398(3)–3.760(3) Å, values similar to those seen in complexes **1**–**4** and in line with Harder’s [3.192(5)–3.386(3) and 3.486(3)–3.930(3) Å for Rb and Cs, respectively]^10h^ and Hill’s [3.264(2)–3.481(2) and 3.411(3)–3.623(2) Å for Rb and Cs, respectively]^10j^ alkali metal Al(I) complexes.

**Figure 6:**
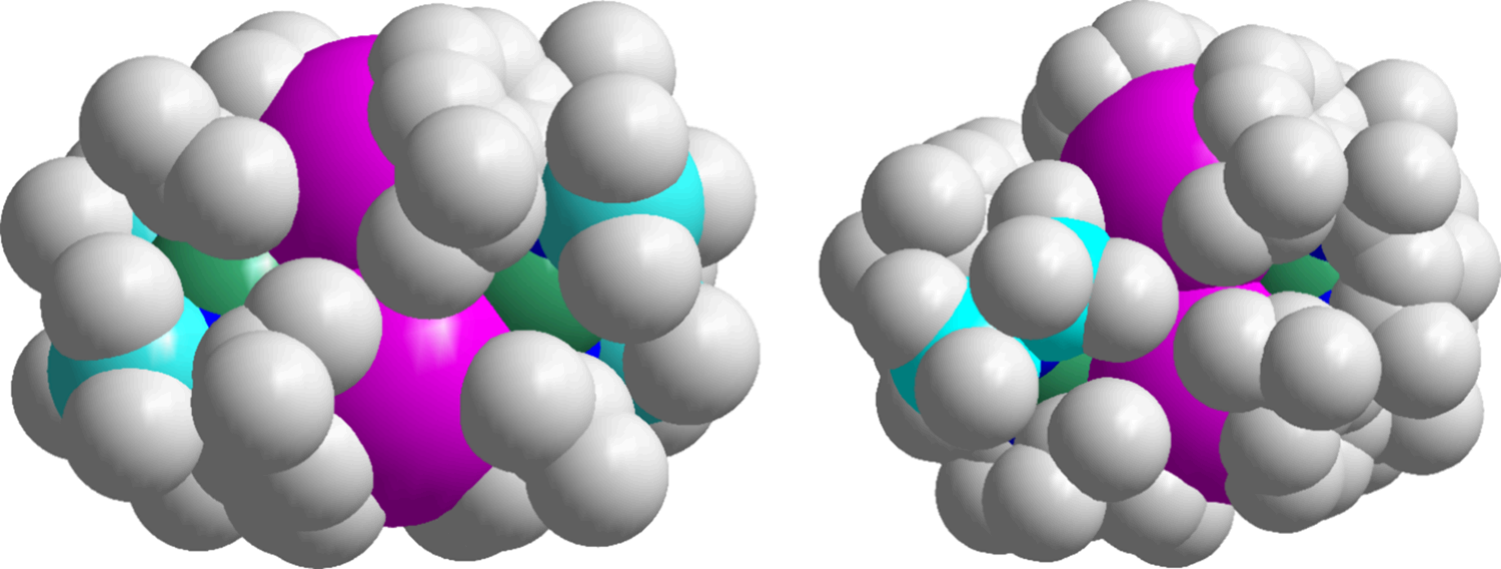
Space filling diagrams of complexes **6** (left) and **E** (right), with pink Cs atoms and green Mg atoms.

As mentioned previously, only **5** could be repeatably synthesized and thus characterized by NMR spectroscopy. The ^1^H NMR spectrum was largely as anticipated, with the key regions being the loss of CH_2_SiMe_3_ resonances from the starting material **3** and the gain of a new resonance at 2.62 ppm, representing the new hydrido ligands in **5**. The inability to isolate a tangible amount of complex **6** hints at a low stability, bucking the trend witnessed in Jones’ alkali metal magnesiate family which show increased thermal stability as group 1 is descended.^18^ This emphasizes the need for the alkali metals to be studied as individual entities and not to be grouped together. We note the different coordination numbers for the Mg center in complexes **5** and **6** versus their related complexes **D** and **E** (3 v 4 coordination), and this may have some role to play in the different stabilities, although there must be other factors at play given that **5** is more stable than **6** despite being isostructural. A comparison of the space filling diagrams of **6** and **E** (Figure 6) suggests that the metal centers in **6** are more exposed than in **E** on account of the lower coordination number and smaller ligands surrounding the [MgHCs]_2_ core.

## Experimental Section

### General Experimental

Due to the air-sensitive nature of s-block organometallic compounds, all manipulations were performed under dry argon or nitrogen using standard Schlenk-line techniques, or in a conventional nitrogen-filled or argon-filled glovebox. Starting materials and research chemicals were obtained from commercial suppliers where appropriate and used without further purification. MgN′_2_,^11^*n*BuMg*N*′,^11^ Rb(CH_2_SiMe_3_),^13c^ Cs(CH_2_SiMe_3_),^13c^ RbN′,^9^ and CsN′^9^ were synthesized according to literature procedures. Hexane was dried by heating to reflux over sodium benzophenone ketyl and then distilled under nitrogen prior to use. Benzene and toluene were degassed with nitrogen, dried over activated aluminum oxide (Innovative Technology, Pure Solv 400–4-MD, Solvent Purification System), and then stored under inert atmosphere over activated 4 Å molecular sieves. Benzene*-d*_6_ and THF*-d*_*8*_ were degassed by freeze–pump–thaw methods and stored over activated 4 Å molecular sieves. NMR spectra were recorded on a Bruker AV3 or AV 400 MHz spectrometer operating at 400.13 MHz for ^1^H and 100.62 MHz for ^13^C. All ^13^C spectra were proton decoupled. ^1^H and ^13^C{^1^H} chemical shifts are expressed in parts per million (δ, ppm) and referenced to residual solvent peaks. Coupling constants (*J*) are given in Hertz (Hz). Elemental analysis was conducted by the Elemental Analysis Service at London Metropolitan University.

### Preparation of [RbMgN′_2_*n*Bu]_∞_ (**1**)

RbN′ (0.334 g, 1.00 mmol) was added in one portion to a colorless solution of *n*BuMgN′ (0.330 g, 1.00 mmol) in toluene (10 mL). After an hour of stirring at room temperature, the sample was kept at −26 °C for 2 weeks which resulted in the deposition of a small number of crystals which were suitable for single-crystal XRD.

### Preparation of [CsMgN′_2_*n*Bu]_∞_ (**2**)

CsN′ (0.381 g, 1.00 mmol) was added in one portion to a colorless solution of *n*BuMgN′ (0.330 g, 1.00 mmol) in toluene (10 mL). After an hour of stirring at room temperature, the sample was kept at −26 °C for 2 weeks which resulted in the deposition of a small number of crystals which were suitable for single-crystal XRD.

### Preparation of [RbMgN′_2_CH_2_SiMe_3_]_∞_ (**3**)

Rb(CH_2_SiMe_3_) (0.173 g, 1.00 mmol) was added in one portion to a colorless solution of MgN′_2_ (0.521 g, 1.00 mmol) in C_6_H_6_ (12 mL) to give a pale-yellow solution. Stirring the mixture at room temperature for 5 min resulted in the precipitation of a white solid. After stirring the mixture for a further 60 min, the precipitate was collected via filtration and subsequently washed with C_6_H_6_ (2 × 5 mL). Drying at high vacuum, the desired compound was obtained as a white solid in 80% (0.578 g, 0.798 mmol) yield. Crystals suitable for single-crystal X-ray diffraction analysis were grown by slowly cooling a hot benzene solution to room temperature.

^1^H NMR (400 MHz, C_6_D_6_:THF-*d*_8_ (4:1), 25 °C): δ = 7.04 (d, ^3^*J*_HH_ = 7.5 Hz, 4H, Ar–C*H*), 6.86 (t, ^3^J_HH_ = 7.5 Hz, 1H, Ar–C*H*), 6.52 (t, ^3^*J*_HH_ = 7.5 Hz, 1H, Ar–C*H*), 4.23–3.89 (m, 4H, C*H*(CH_3_)_2_), 1.38–1.24 (m, 18H, CH(C*H*_*3*_)_2_), 1.14 (d, ^3^*J*_HH_ = 6.9 Hz, 6H, CH(C*H*_*3*_)_2_), 0.36 (s, 9H, Si(C*H*_*3*_)_3_), 0.24 (s, 9H, Si(C*H*_*3*_)_3_), 0.23 (s, 9H, Si(C*H*_*3*_)_3_), −1.44 (s, 2H, C*H*_*2*_-Si(CH_3_)_3_).

^13^C{^1^H} NMR (100 MHz, C_6_D_6_:THF-*d*_*8*_ (4:1), 25 °C): δ = 156.6 (*C*_arom_), 153.0 (*C*_arom_), 145.3 (*C*_arom_), 140.4 (*C*_arom_H), 123.3 (*C*_arom_), 122.6 (*C*_arom_), 119.3 (*C*_arom_), 110.7 (*C*_arom_H), 27.3 (*C*_aliph_H), 27.0 (*C*_aliph_H), 25.4 (*C*_aliph_H_3_), 25.4 (*C*_aliph_H_3_), 24.7 (*C*_aliph_H_3_), 5.4 (Si(*C*H_3_)_3_), 4.7 (Si(*C*H_3_)_3_), 3.9 (Si(*C*H_3_)_3_), −7.0 (*C*H_2_–Si(CH_3_)_3_).

#### Elemental Analysis

Calculated values for C_34_H_63_MgN_2_RbSi_3_ (693.92 g/mol): C 58.85, H 9.15, N 4.04; found (average of 3 samples measurements): C 58.78, H 9.00, N 3.74.

### Preparation of [CsMgN′_2_CH_2_SiMe_3_]_∞_ (**4**)

Cs(CH_2_SiMe_3_) (0.291 g, 1.38 mmol) was added in one portion to a vigorously stirred, colorless solution of MgN′_2_ (0.721 g, 1.38 mmol) in C_6_H_6_ (20 mL) to give a slightly yellow solution. Stirring the solution at room temperature for 2 h resulted in the precipitation of a white solid. After stirring the mixture for further 3 h, the precipitate was collected via filtration and subsequently washed with C_6_H_6_ (2 × 5 mL). Drying at high vacuum, the desired compound was obtained as a white solid in 62% (0.663 g, 0.859 mmol) yield. Crystals suitable for single-crystal X-ray diffraction analysis were grown by slowly cooling a hot benzene solution to room temperature.

^1^H NMR (400 MHz, C_6_D_6_:THF-*d*_8_ (4:1), 25 °C): δ = 7.07 (d, ^3^*J*_HH_ = 7.5 Hz, 4H, Ar–C*H*), 6.89 (t, ^3^J_HH_ = 7.4 Hz, 1H, Ar–C*H*), 6.56 (t, ^3^*J*_HH_ = 7.4 Hz, 1H, Ar–C*H*), 4.11 (sept, ^3^*J*_HH_ = 6.8 Hz, 2H, C*H*(CH_3_)_2_), 3.90 (sept, ^3^*J*_HH_ = 6.8 Hz, 2H, C*H*(CH_3_)_2_), 1.34 (d, ^3^*J*_HH_ = 6.9 Hz, 6H, CH(C*H*_*3*_)_2_), 1.28 (d, ^3^*J*_HH_ = 6.9 Hz, 12H, CH(C*H*_*3*_)_2_), 1.16 (d, ^3^*J*_HH_ = 6.9 Hz, 6H, CH(C*H*_*3*_)_2_), 0.41 (s, 9H, Si(C*H*_*3*_)_3_), 0.27 (s, 9H, Si(C*H*_*3*_)_3_), 0.26 (s, 9H, Si(C*H*_*3*_)_3_), −1.41 (s, 2H, C*H*_*2*_-Si(CH_3_)_3_).

^13^C{^1^H} NMR (100 MHz, C_6_D_6_:THF-*d*_*8*_ (4:1), 25 °C): δ = 156.1 (*C*_arom_), 153.0 (*C*_arom_), 145.3 (*C*_arom_), 140.4 (*C*_arom_H), 123.3 (*C*_arom_), 122.8 (*C*_arom_), 119.4 (*C*_arom_), 110.5 (*C*_arom_H), 27.4 (*C*_aliph_H), 27.0 (*C*_aliph_H), 25.6 (*C*_aliph_H_3_), 25.5 (*C*_aliph_H_3_), 24.7 (*C*_aliph_H_3_), 5.6 (Si(*C*H_3_)_3_), 4.8 (Si(*C*H_3_)_3_), 3.9 (Si(*C*H_3_)_3_), −7.0 (*C*H_2_–Si(CH_3_)_3_).

#### Elemental Analysis

Calculated values for C_34_H_63_MgCsN_2_Si_3_ (741.36 g/mol): C 55.08, H 8.57 N 3.78; found (average of 3 samples measurements): C 54.70, H 8.47, N 3.66.

### Preparation of [RbMgN′_2_H]_2_ (**5**)

In a 25 mL J. Young ampule, 1,4-cyclohexadiene (210 μL, 2.25 mmol, 3.00 equiv) was added to a suspension of [RbMgN′_2_CH_2_SiMe_3_]_∞_ (520 mg, 0.75 mmol) in benzene (10 mL) at room temperature. The resulting white reaction mixture was heated at 50 °C for 90 min to give a colorless solution. Small amounts of black, insoluble material were removed by filtration, and all volatiles were removed under reduced pressure. The resulting white solid was washed with *n*-hexane (2 × 3 mL). Drying at high vacuum, the desired compound was obtained as a white solid in 65% (295 mg, 0.485 mmol) yield. Crystals suitable for single-crystal X-ray diffraction analysis were grown by slow evaporation from a benzene solution at room temperature.

^1^H NMR (400 MHz, C_6_D_6_, 25 °C): δ = 6.84 (d, ^3^*J*_HH_ = 7.2 Hz, 8H, Ar–C*H*), 6.77–6.68 (m, 4H, Ar–C*H*), 4.01 (sept, ^3^*J*_HH_ = 6.8 Hz, 8H, C*H*(CH_3_)_2_), 2.62 (s, 2H, Mg*H*), 1.29 (d, ^3^*J*_HH_ = 6.9 Hz, 24H, CH(C*H*_*3*_)_2_), 0.85 (d, ^3^*J*_HH_ = 6.9 Hz, 24H, CH(C*H*_*3*_)_2_), 0.36 (s, 36H, Si(C*H*_*3*_)_3_).

^13^C{^1^H} NMR (100 MHz, C_6_D_6_, 25 °C): δ = 155.0 (*C*_arom_), 147.0 (*C*_arom_), 122.7 (*C*_arom_H), 120.2 (*C*_arom_H), 27.3 (*C*_aliph_H), 25.2 (*C*_aliph_H_3_), 23.4 (*C*_aliph_H_3_), 4.0 (Si(*C*H_3_)_3_).

#### Elemental Analysis

Calculated values for C_60_H_106_Rb_2_Mg_2_N_4_Si_4_ (1215.42 g/mol): C 59.29, H 8.79 N 4.61; found (average of 3 samples measurements): C 58.71, H 8.52, N 4.17.

### Preparation of [CsMgN′_2_H]_2_ (**6**)

[CsMgN′_2_CH_2_SiMe_3_]_∞_ (556 mg, 0.75 mmol) was suspended in benzene (10 mL), and 1,4-cyclohexadiene (210 μL, 2.25 mmol, 3.00 equiv) was added. The suspension was stirred at 50 °C until all solid dissolved (3 h). After removal of all volatiles in high vacuum, the remaining crude solid was dissolved in hexane and left to slowly evaporate at room temperature. This resulted in the isolation of a few single crystals, but the method proved unrepeatable despite several attempts and so no solution data could be obtained.

### Crystallography

Crystallographic data for new complexes **1–6** were collected on an Oxford Diffraction Gemini S instrument with graphite-monochromated Mo–Kα (λ 0.71073 Å) radiation or on Rigaku XtaLAB Synergy-S with monochromated Cu–Kα (λ 1.54184 Å) radiation. The measured data were processed with the CrysAlisPro^19^ software package. Using Olex2,^20^ the structure was solved with the ShelXT^21^ structure solution program using intrinsic phasing and refined with the SHELXL^22^ refinement package using least-squares minimization or by the full-matrix least-squares method using SHELXL-2018 implemented within WINGX.^21^ All nonhydrogen atoms were refined using anisotropic thermal parameters unless noted otherwise. Table 3 contains selected data and refinement details.

**Table 3:** Crystallographic Data and Refinement Details for Complexes **1**–**6**

	1	2	3	4	5	6
empirical formula	C_37_H_61_MgN_2_RbSi_2_	C_37_H_68_CsMgN_2_Si_2_	C_34_H_63_MgN_2_RbSi_3_	C_34_H_63_CsMgN_2_Si_3_	C_60_H_106_Mg_2_N_4_Rb_2_Si_4_	C_60_H_106_Cs_2_Mg_2_N_4_Si_4_
MW	702.86	754.33	693.91	741.35	1215.40	1310.28
crystal system	monoclinic	monoclinic	monoclinic	monoclinic	monoclinic	monoclinic
space group	I 2/a	P 2_1_/c	*P* 2_1_/*n*	*P*2_1_/*n*	*P*2_1_/*n*	*P*2_1_/*n*
a/Å	18.5223(6)	9.5256(5)	11.5322(9)	11.5881(2)	12.5461(1)	12.3828(1)
b/Å	11.8791(3)	21.8810(6)	17.9404(8)	18.0853(3)	18.0181(2)	18.3471(2)
c/Å	36.8177(11)	20.1157(8)	19.9088(13)	19.9505(4)	31.6770(2)	31.7569(2)
α/deg	90	90	90	90	90	90
β/deg	90.282(3)	90.808(4)	102.492(7)	102.513(2)	92.219(1)	92.038(1)
γ/deg	90	90	90	90	90	90
V/Å^3^	8100.8(4)	4192.3(3)	4021.5(5)	4081.80(13)	7155.43(11)	7210.24(11)
*Z*	8	4	4	4	4	4
ρ/g cm^–3^	1.153	1.195	1.146	1.206	1.128	1.207
reflns measured	42818	41691	41542	45162	157857	156057
unique reflns	8617	8191	7928	9844	18029	18156
*R*_int_	0.0872	0.1049	0.1263	0.0542	0.0398	0.0485
obs. reflns [*I* > 2σI]	5854	5562	4496	7205	14867	15362
GooF	1.022	1.112	1.010	1.060	1.025	1.257
*R*	0.0574	0.0901	0.0599	0.0414	0.0331	0.0470
ω*R*	0.0971	0.1318	0.1328	0.0708	0.0474	0.0605
largest diff peak/hole eÅ^–3^	0.484/–0.332	1.491/–0.950	0.615/–0.444	0.596/–0.451	0.401/–0.353	0.787/–1.137

## Conclusions

Motivated by the paucity of knowledge of the organometallic chemistry of the heavier alkali metals rubidium and cesium, this study reports six new compounds in this category. Well-established for the lighter alkali metals, cocomplexation proved an equally effective method for synthesizing bis(amido)alkyl magnesiates of rubidium and cesium. All four target compounds were prepared and crystallographically characterized, but the reactions involving the CH_3_CH_2_CH_2_CH_2_ alkyl products were not satisfactory, only producing poor yields of the desired magnesiates, and thus necessitated a switch to the silyl-stabilized alkyl group Me_3_SiCH_2_ for cleaner reactions and full characterization of the isolable products. As expected, the softer components of the ligands, the π-electron cloud of the N(SiMe_3_)(Dipp) amido ligand and the alpha-C atom of the alkyl ligand, stabilize rubidium and cesium centers, while the harder amido N centers bind only to magnesium. Molecular hydrides of rubidium and cesium are exceptionally rare so the fact that both hydrido-magnesiates [(AM)MgN′_2_H]_2_ could be accessed by reaction of the [(AM)MgN′_2_CH_2_SiMe_3_]_∞_ complexes with 1,4-cyclohexadiene and crystallographically characterized is promising even though the cesium complex could not be reproduced. This may suggest that the amido ligand used here has sufficient steric and electronic coordinative capacity to stabilize rubidium but not cesium. Arguably, the key conclusion from this study is that it emphasizes that these heavier alkali metals should not be classed together as mere gegenions in organoelement chemistry, as important but subtle distinctions may give rise to contrasting performances in future applications in homogeneous catalysis, an area we are currently exploring.

## Supporting Information Available

NMR spectra of complexes 3–5 (PDF)

## Notes

A data set containing the raw crystallographic (cif) and NMR data can be found at 10.15129/df9709a6-4985-45dd-bd9f-2d7d8faa180d

## References

[ref1] aMulvey R. E.; Mongin F.; Uchiyama M.; Kondo Y. Deprotonative Metalation Using Ate Compounds: Synergy, Synthesis, and Structure Building. Angew. Chem., Int. Ed. 2007, 46, 3802–3824. 10.1002/anie.200604369. 17444540

[ref2] aRoy M. M. D.; Omaña A. A.; Wilson A. S. S.; Hill M. S.; Aldridge S.; Rivard E. Molecular Main Group Metal Hydrides. Chem. Rev. 2021, 121, 12784–12965. 10.1021/acs.chemrev.1c00278. 34450005

[ref3] aGentner T. X.; Mulvey R. E. Alkali-Metal Mediation: Diversity of Applications in Main-Group Organometallic Chemistry. Angew. Chem., Int. Ed. 2021, 60, 9247–9262. 10.1002/anie.202010963. PMC8247348 33017511

[ref4] Pearce K. G.; Liu H.-Y.; Neale S. E.; Goff H. M.; Mahon M. F.; McMullin C. L.; Hill M. S. Alkali Metal Reduction of Alkali Metal Cations. Nat. Commun. 2023, 14, 8147 10.1038/s41467-023-43925-5. 38065953 PMC10709313

[ref5] https://www.euchems.eu/euchems-periodic-table/ accessed April 16, 2024.

[ref6] aGentner T. X.; Kennedy A. R.; Hevia E.; Mulvey R. E. Alkali Metal (Li, Na, K, Rb, Cs) Mediation in Magnesium Hexamethyldisilazide [Mg(HMDS)_2_] Catalysed Transfer Hydrogenation of Alkenes. ChemCatChem. 2021, 13, 2371–2378. 10.1002/cctc.202100218.

[ref7] Bauer H.; Thum K.; Alonso M.; Fischer C.; Harder S. Alkene Transfer Hydrogenation with Alkaline-Earth Metal Catalysts. Angew. Chem., Int. Ed. 2019, 58, 4248–4253. 10.1002/anie.201813910. 30667149

[ref8] aKennepohl D. K.; Brooker S.; Sheldrick G. M.; Roesky H. W. Synthesis and Molecular Structure of the Solvent-Free [LiN(SiMe_3_)(2,6*i*Pr_2_C_6_H_3_)]_2_ Dimer. Chem. Ber. 1991, 124, 2223–2225. 10.1002/cber.19911241013.

[ref9] Ballmann G. M.; Gentner T. X.; Kennedy A. R.; Hevia E.; Mulvey R. E. Heavy Alkali Metal Manganate Complexes: Synthesis, Structures and Solvent-Induced Dissociation Effects. Chem.—Eur. J. 2022, 28, e202201716 10.1002/chem.202201716. 35775467 PMC9804227

[ref10] aHicks J.; Vasko P.; Goicoechea J. M.; Aldridge S. Synthesis, Structure and Reaction Chemistry of a Nucleophilic Aluminyl Anion. Nature 2018, 557, 92–95. 10.1038/s41586-018-0037-y. 29662211

[ref11] Vargas W.; Englich U.; Ruhlandt-Senge K. A Novel Group of Alkaline Earth Metal Amides: Syntheses and Characterization of M[N(2,6-^*i*^Pr_2_C_6_H_3_)(SiMe_3_)]_2_(THF)_2_ (M = Mg, Ca, Sr, Ba) and the Linear, Two-Coordinate Mg[N(2,6-^*i*^Pr_2_C_6_H_3_)(SiMe_3_)]_2_. Inorg. Chem. 2002, 41, 5602–5608. 10.1021/ic0203668. 12377060

[ref12] aGilman H.; Beel J. A.; Brannen C. G.; Bullock M. W.; Dunn G. E.; Miller L. S. The Preparation of *n*-Butyllithium. J. Am. Chem. Soc. 1949, 71, 1499–1500. 10.1021/ja01172a510.

[ref13] aTatic T.; Ott H.; Stalke D. Deaggregation of Trimethylsilylmethyllithium. Eur. J. Inorg. Chem. 2008, 2008, 3765–3768. 10.1002/ejic.200800610.

[ref14] aMcMullen J. S.; Huo R.; Vasko P.; Edwards A. J.; Hicks J. Anionic Magnesium and Calcium Hydrides: Transforming CO into Unsaturated Disilyl Ethers. Angew. Chem., Int. Ed. 2023, 62, e202215218 10.1002/anie.202215218. PMC10100151 36344462

[ref15] Jackson R. A.; Matthews A. J. R.; Vasko P.; Mahon M. F.; Hicks J.; Liptrot D. J. An Acyclic Aluminyl Anion. Chem. Commun. 2023, 59, 5277–5280. 10.1039/D3CC01317K. 37060116

[ref16] aGallagher D. J.; Henderson K. W.; Kennedy A. R.; O’Hara C. T.; Mulvey R. E.; Rowlings R. B. Hydride Encapsulation in s-Block Metal Inverse Crown Chemistry Chem. Commun. 2002, 376–377 10.1039/B110117J. 12120082

[ref17] Liptrot D. J.; Hill M. S.; Mahon M. F. Heterobimetallic s-Block Hydrides by σ-Bond Metathesis. Chem.—Eur. J. 2014, 20, 9871–9874. 10.1002/chem.201403215. 25042080

[ref18] Evans M. J.; Jones C. Synthesis and Reactivity of Alkali Metal Hydrido-Magnesiate Complexes which Exhibit Group 1 Metal Counter-Cation Specific Stability. Inorg. Chem. 2023, 62, 14393–14401. 10.1021/acs.inorgchem.3c02086. 37602922

[ref19] Dolomanov O. V.; Bourhis L. J.; Gildea R. J.; Howard J. A. K.; Puschmann H. *OLEX2*: A Complete Structure Solution, Refinement and Analysis Program. J. Appl. Crystallogr. 2009, 42, 339–341. 10.1107/S0021889808042726.

[ref20] Sheldrick G. M. Crystal Structure Refinement with *SHELXL*. Acta Crystallogr. 2015, C71, 3–8. 10.1107/S2053229614024218. PMC4294323 25567568

[ref21] Sheldrick G. M. A short history of SHELX. Acta Crystallogr. 2008, C64, 112–122. 10.1107/S0108767307043930. 18156677

[ref22] Farrugia L. J. *WinGX* and *ORTEP for Windows*: An Update. J. Appl. Crystallogr. 2012, 45, 849–854. 10.1107/S0021889812029111.

