# Supplementary material for: Application of Bis(amido)alkyl Magnesiates toward the Synthesis of Molecular Rubidium and Cesium Hydrido-magnesiates
Source: Organometallics. 2024 Jun 13;43(12):1393–401. doi: 10.1021/acs.organomet.4c00190 (PMC11200325; doi:10.1021/acs.organomet.4c00190)
Supplement: Supplementary file 1 — om4c00190_si_001.pdf [file om4c00190_si_001.pdf]

Application of bis(amido)alkyl magnesiates towards the synthesis of molecular rubidium and caesium hydrido-magnesiates

Thomas X. Gentner, Gerd M. Ballmann, Sumanta Banerjee, Alan R. Kennedy, Stuart D. Robertson,* and Robert E. Mulvey*

WestChem, Department of Pure and Applied Chemistry, University of Strathclyde, 295 Cathedral Street, Glasgow, G1 1XL, UK.

SUPPORTING INFORMATION

Selected NMR spectra

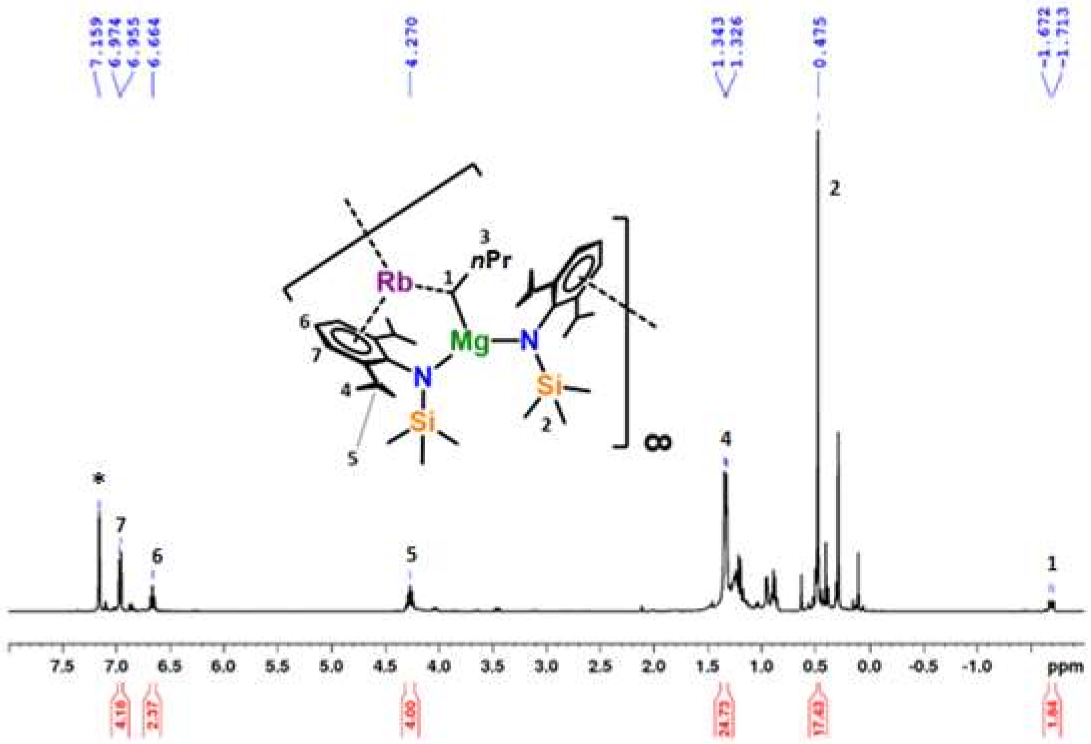

Figure S1 ^{1}H NMR spectrum of $[(Rb)MgN'_2nBu]_\infty$ **1** in C_6D_6. Residual signal for deuterated solvent marked with *

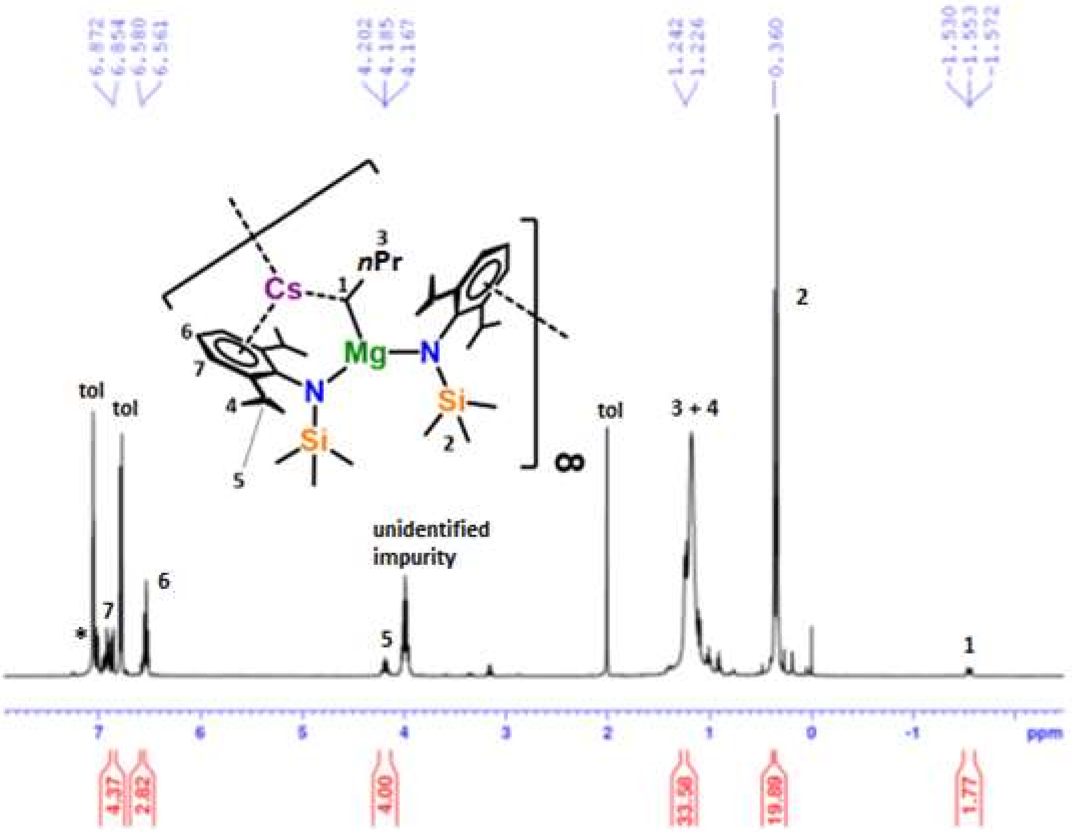

Figure S2 ^{1}H NMR spectrum of $[(Cs)MgN'_2nBu]_\infty$ **2** in C_6D_6. Residual signal for deuterated solvent marked with *

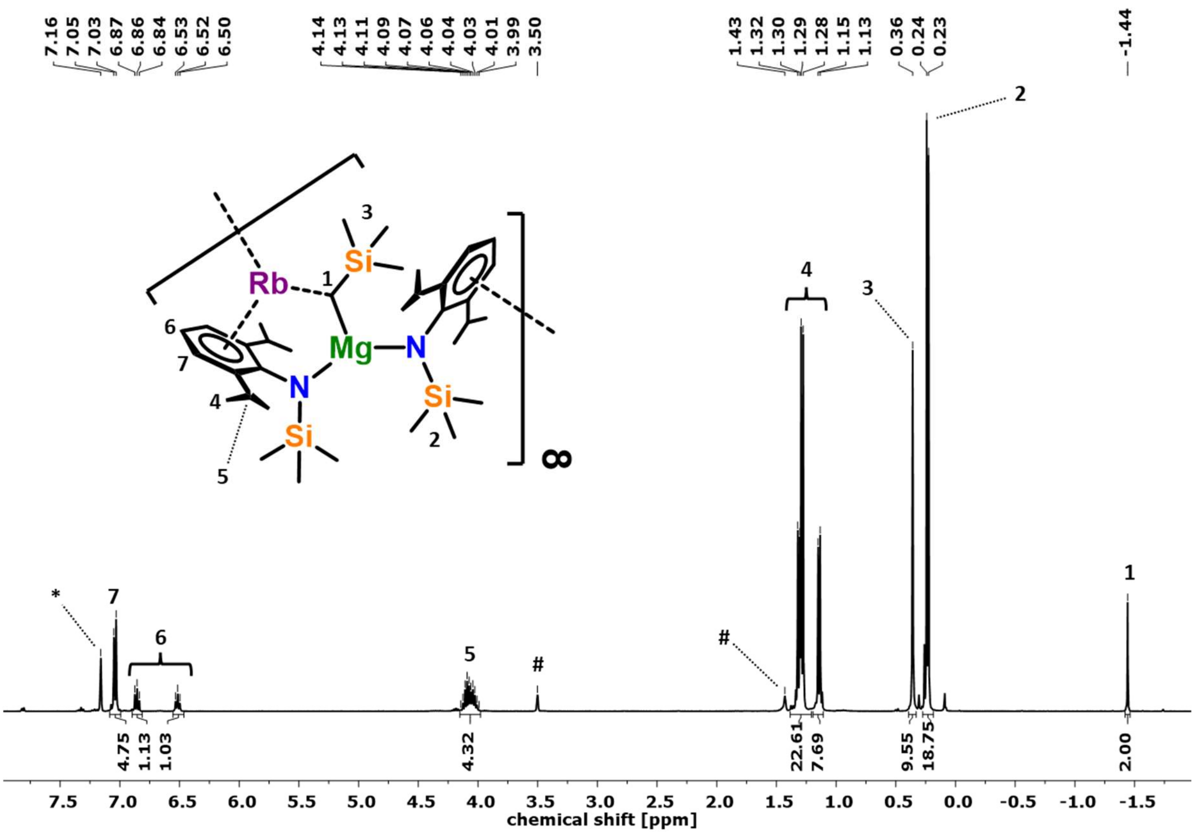

Figure S3 ^{1}H NMR spectrum of $[(Rb)MgN'_2CH_2SiMe_3]_\infty$ **3** in a mixture of benzene-d_6:THF-d_8 (4:1). Residual signal for deuterated solvents marked with * and #.

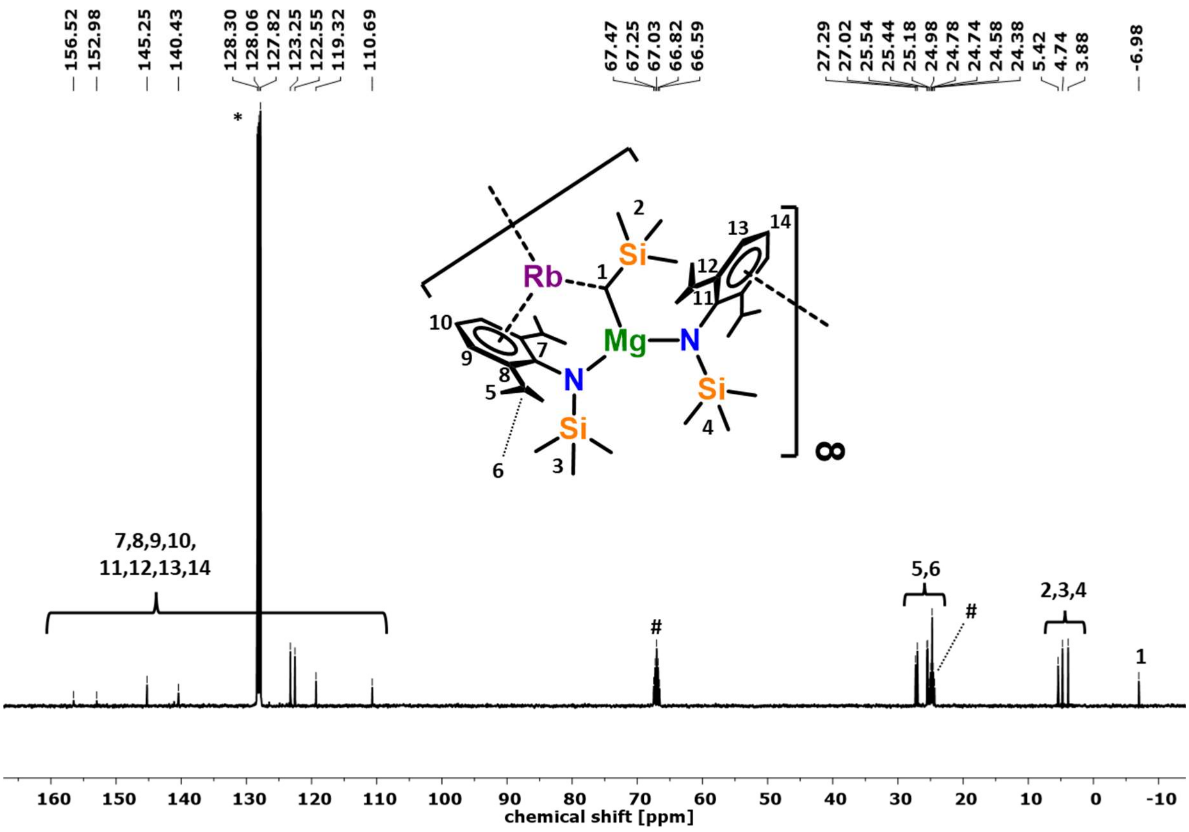

Figure S4 ^{13}C NMR spectrum of $[(Rb)MgN'_2CH_2SiMe_3]_\infty$ **3** in a mixture of benzene-d_6:THF-d_8 (4:1). Residual signal for deuterated solvents marked with * and #.

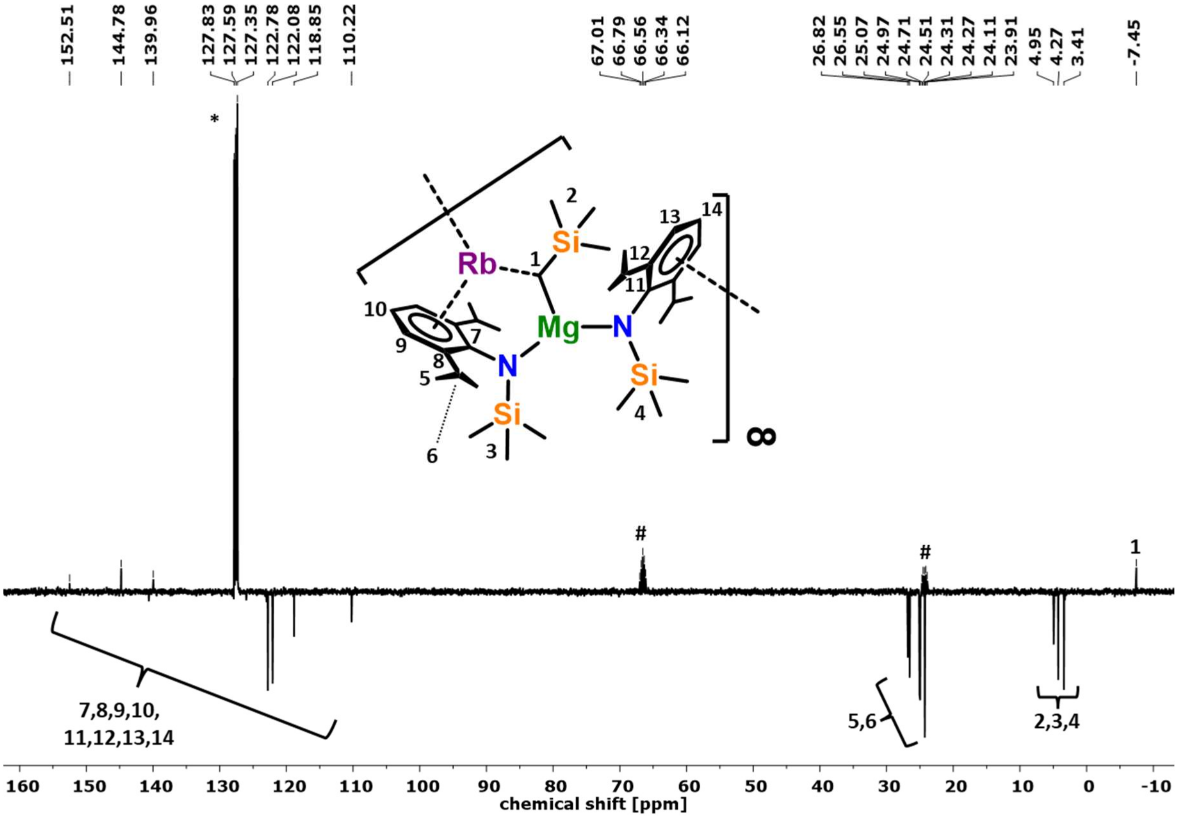

Figure S5 ^{13}C-DEPTQ NMR spectrum of $[(Rb)MgN'_2CH_2SiMe_3]_\infty$ **3** in a mixture of benzene-d_6:THF-d_8 (4:1). Residual signal for deuterated solvents marked with * and #.

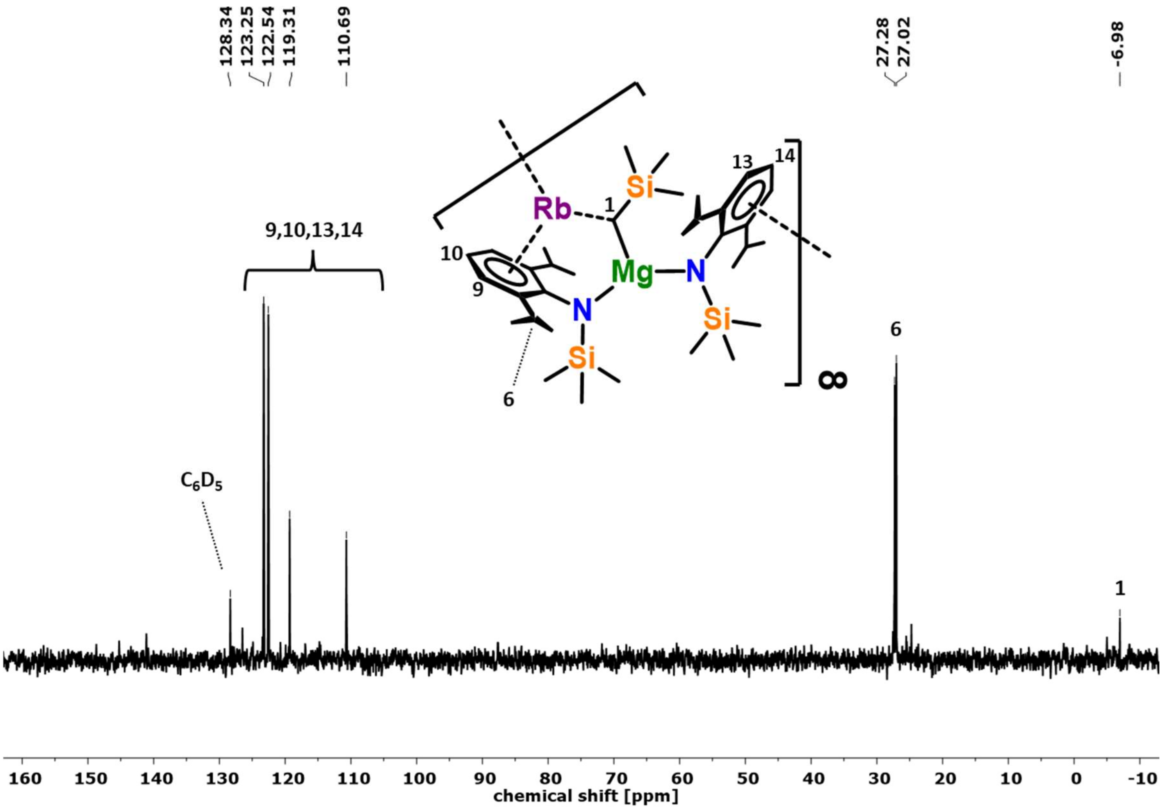

Figure S6 ^{13}C-DEPT-90 NMR spectrum of $[(Rb)MgN'_2CH_2SiMe_3]_\infty$ **3** in a mixture of benzene-d_6:THF-d_8 (4:1).

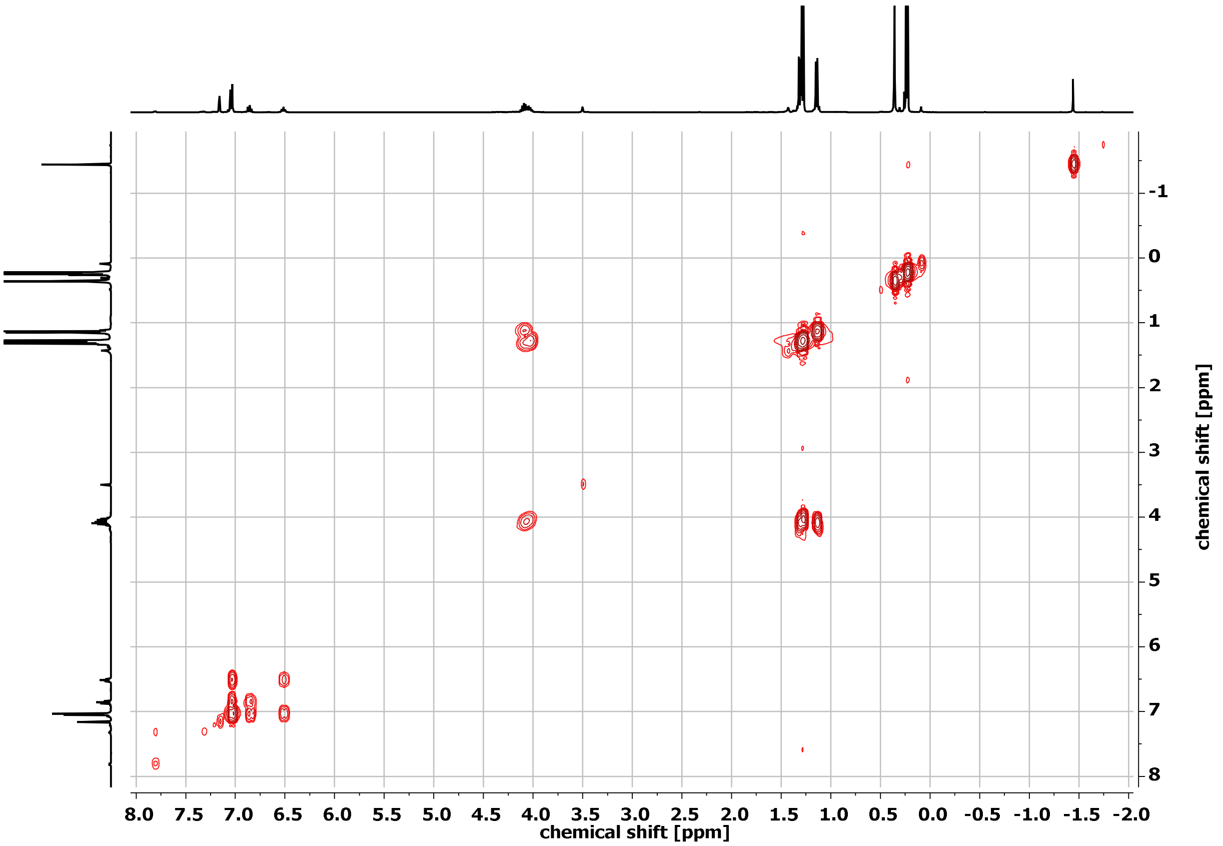

Figure S7 $^{1}H^{1}H$-COSY NMR spectrum of [(Rb)MgN'$_2$CH$_2$SiMe$_3$]$_\infty$ **3** in a mixture of benzene-d_6:THF-d_8 (4:1).

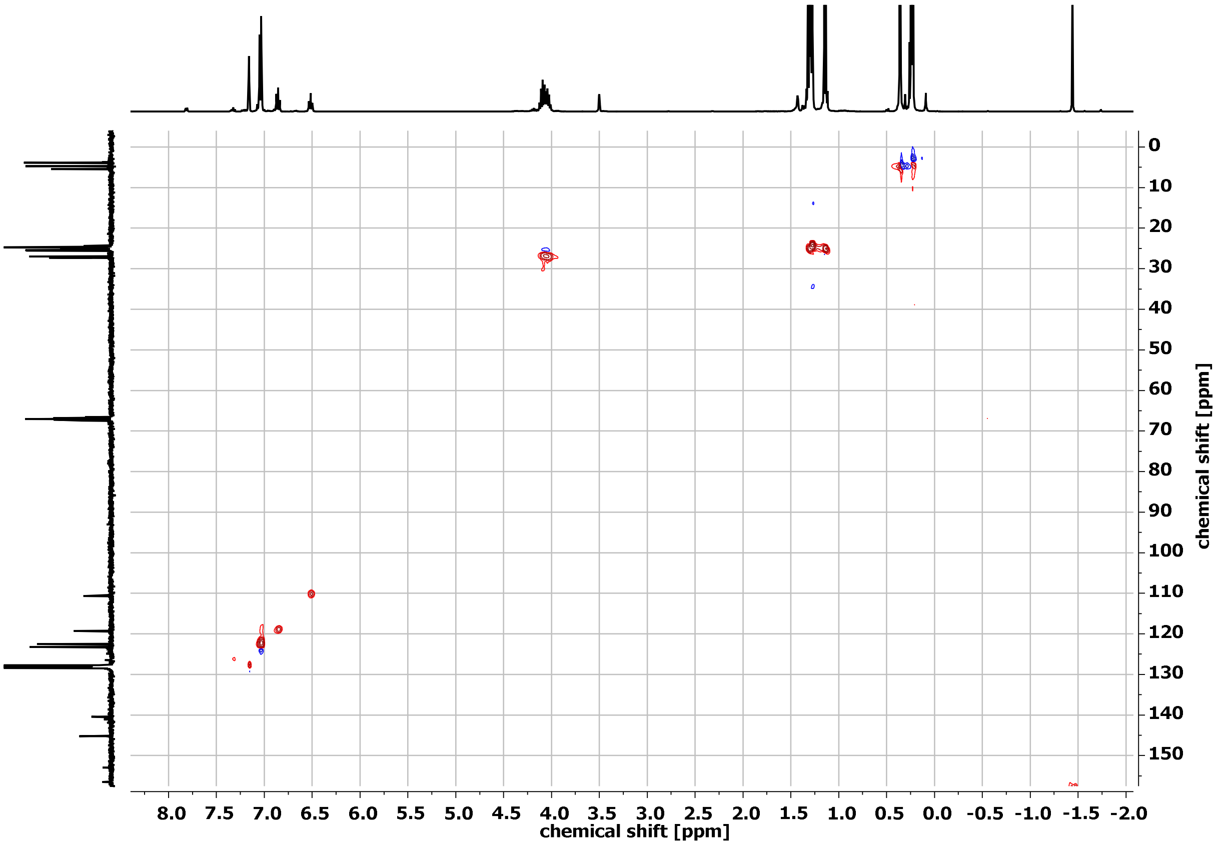

Figure S8 $^{1}H^{13}C$-HSQC NMR spectrum of [(Rb)MgN'$_2$CH$_2$SiMe$_3$]$_\infty$ **3** in a mixture of benzene-d_6:THF-d_8 (4:1).

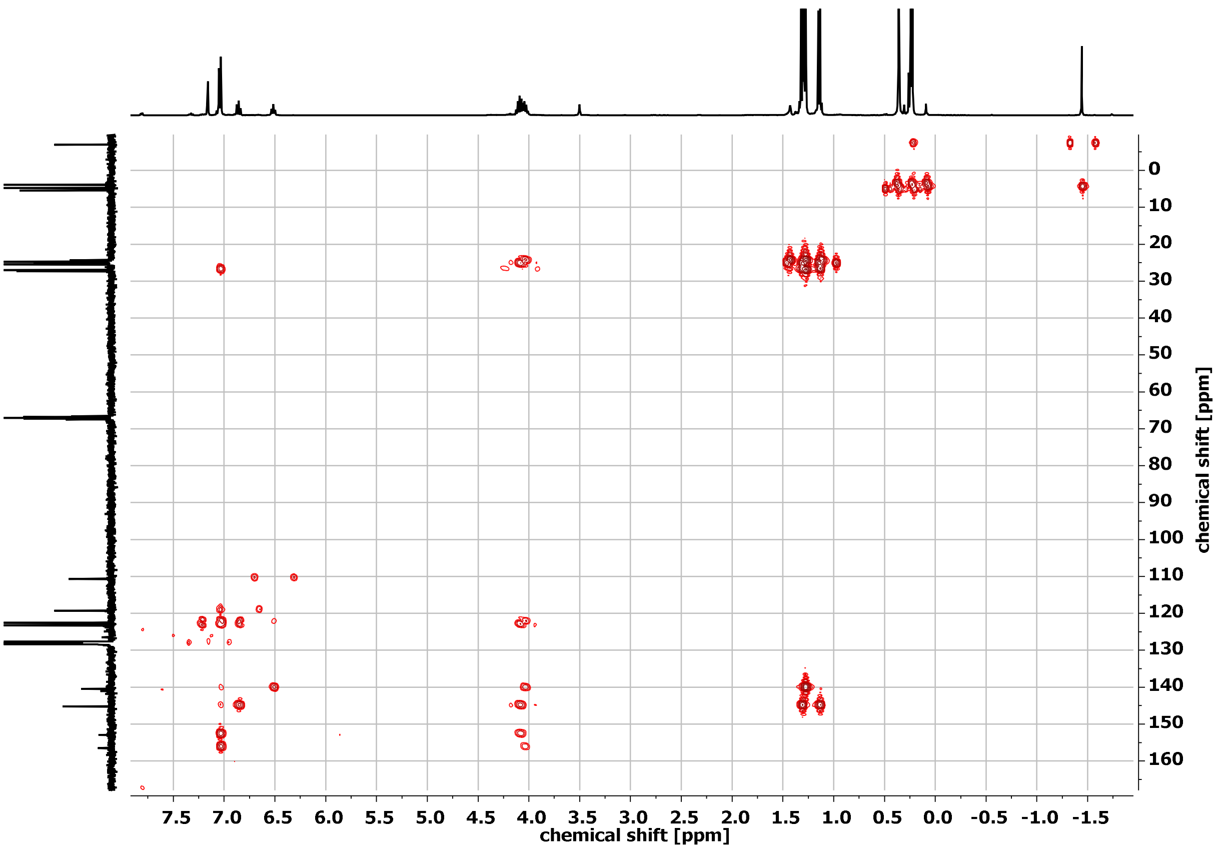

Figure S9 $^{1}H^{13}C$-HMBC NMR spectrum of $[(Rb)MgN'_2CH_2SiMe_3]_\infty$ **3** in a mixture of benzene-*d*$_6$:THF-*d*$_8$ (4:1).

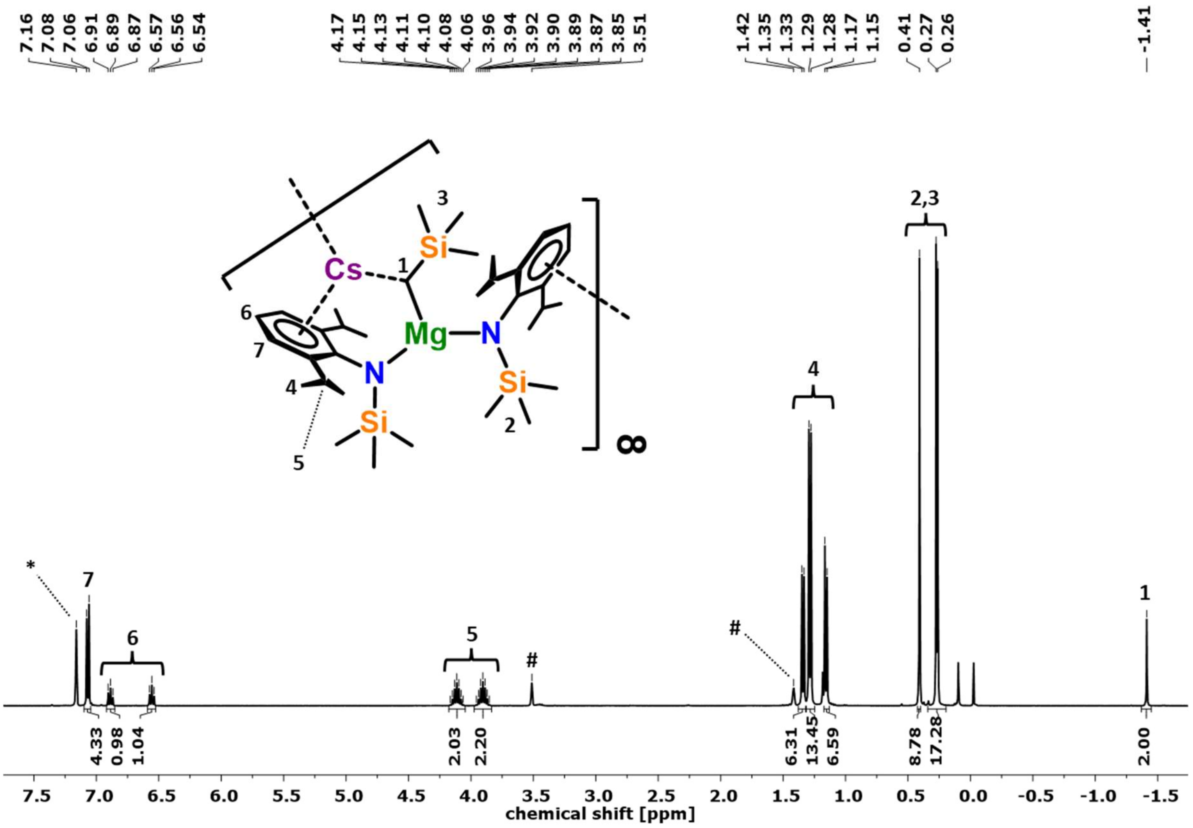

Figure S10 ^{1}H NMR spectrum of $[(Cs)MgN'_2CH_2SiMe_3]_\infty$ **4** in a mixture of benzene-*d*$_6$:THF-*d*$_8$ (4:1). Residual signal for deuterated solvents marked with * and #.

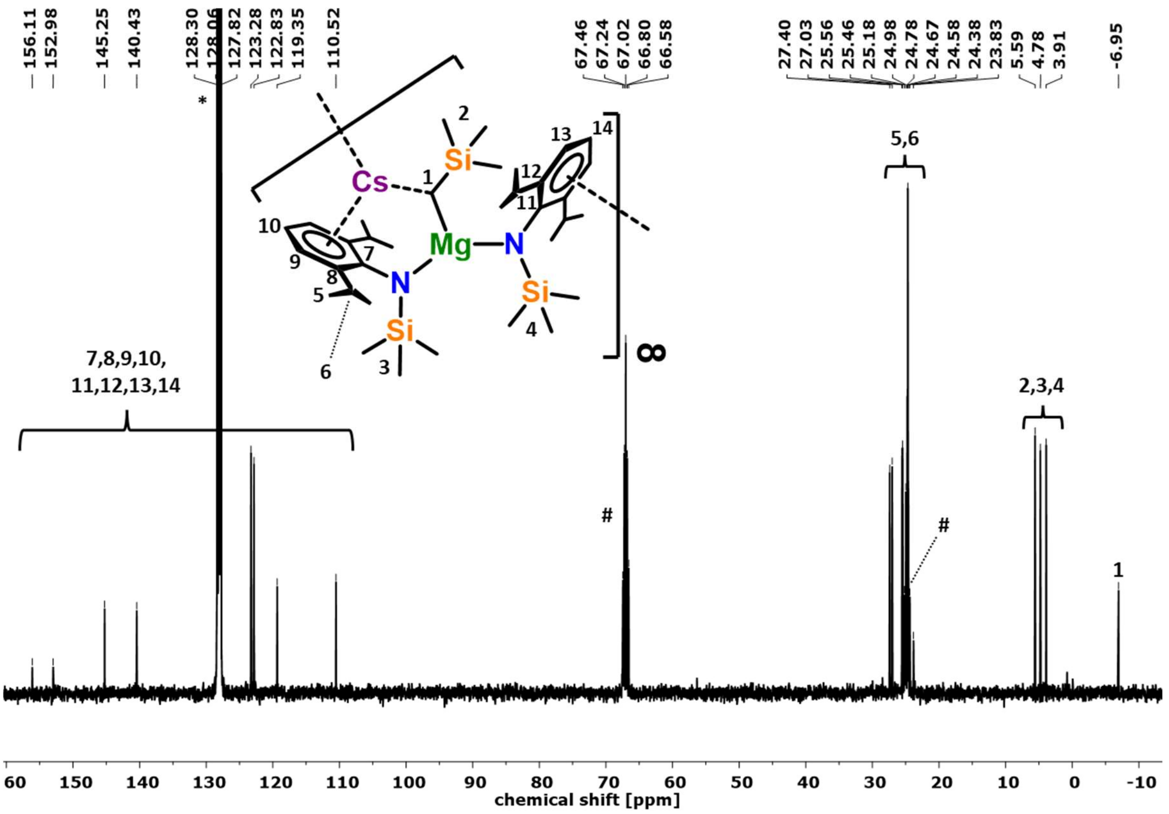

Figure S11 ^{13}C NMR spectrum of $[(Cs)MgN'_2CH_2SiMe_3]_\infty$ **4** in a mixture of benzene-d_6:THF-d_8 (4:1). Residual signal for deuterated solvents marked with * and #.

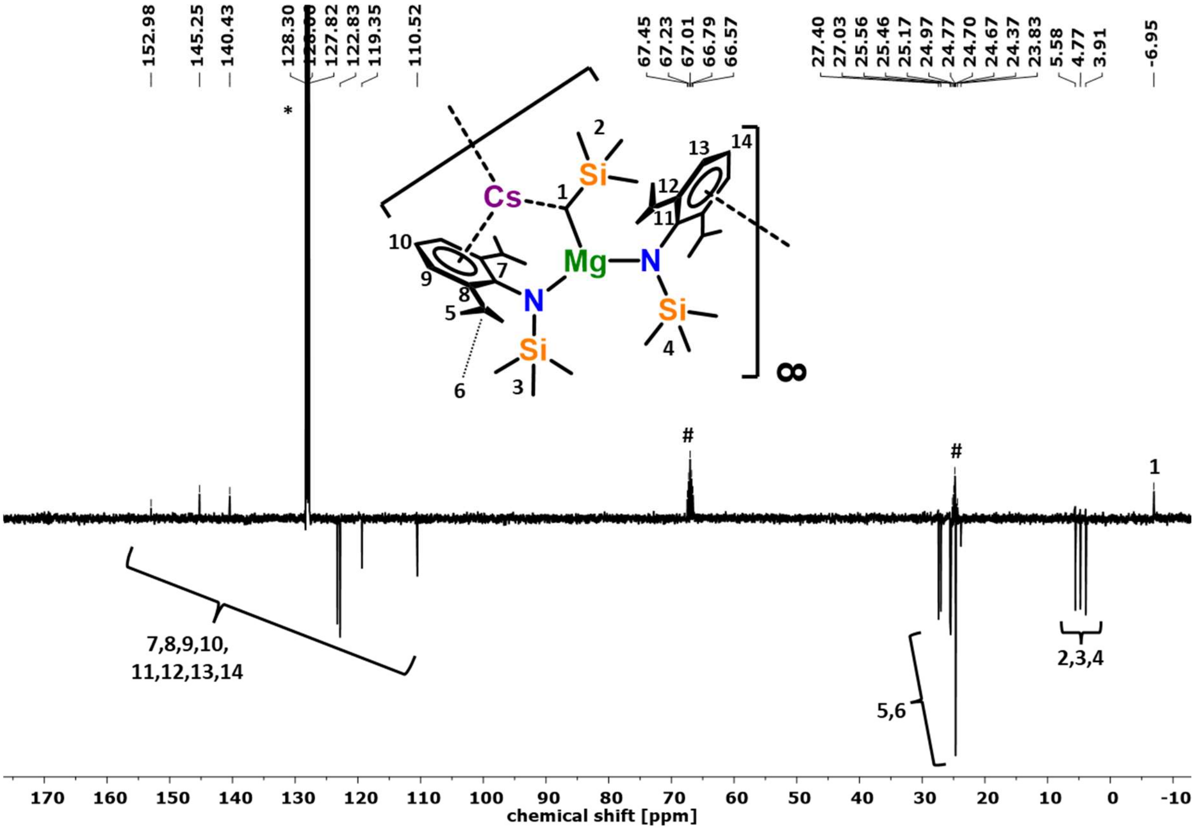

Figure S12 ^{13}C-DEPTQ NMR spectrum of $[(Cs)MgN'_2CH_2SiMe_3]_\infty$ **4** in a mixture of benzene-d_6:THF-d_8 (4:1). Residual signal for deuterated solvents marked with * and #.

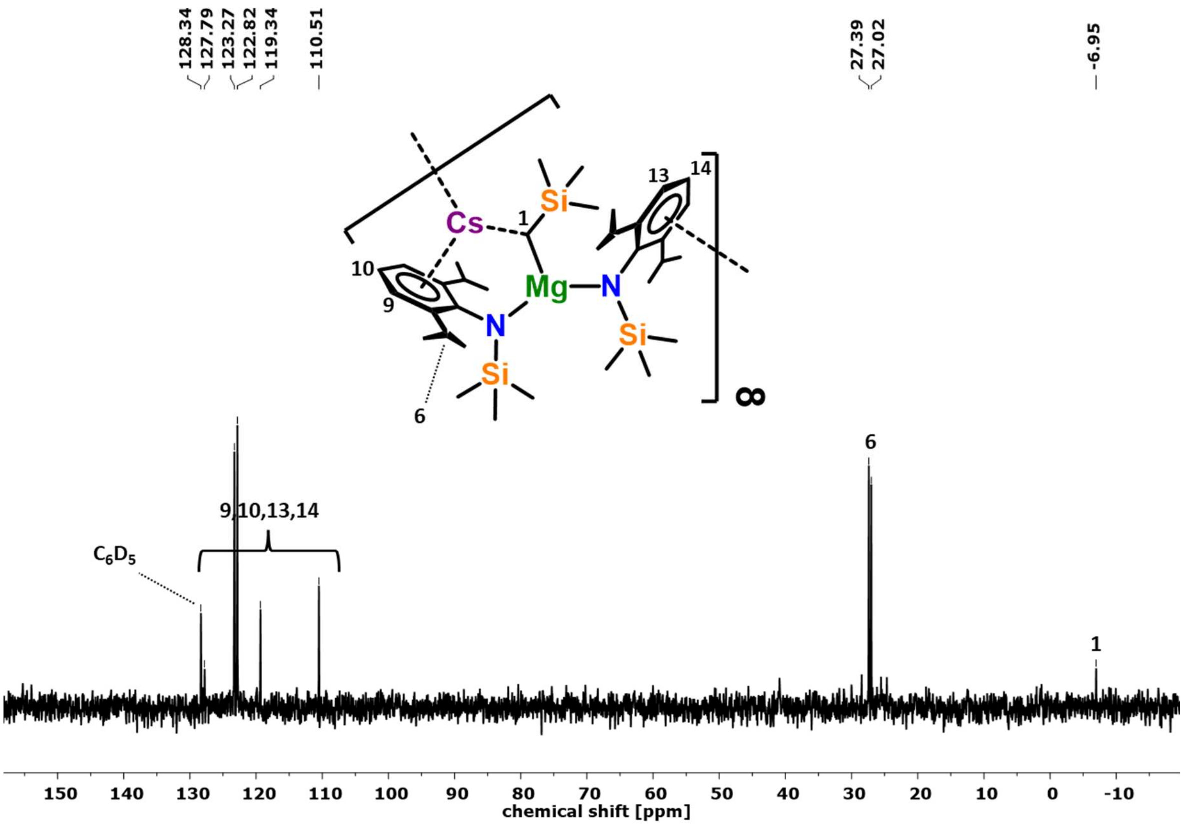

Figure S13 ^{13}C-DEPT-90 NMR spectrum of $[(Cs)MgN'_2CH_2SiMe_3]_\infty$ **4** in a mixture of benzene-*d*$_6$:THF-*d*$_8$ (4:1).

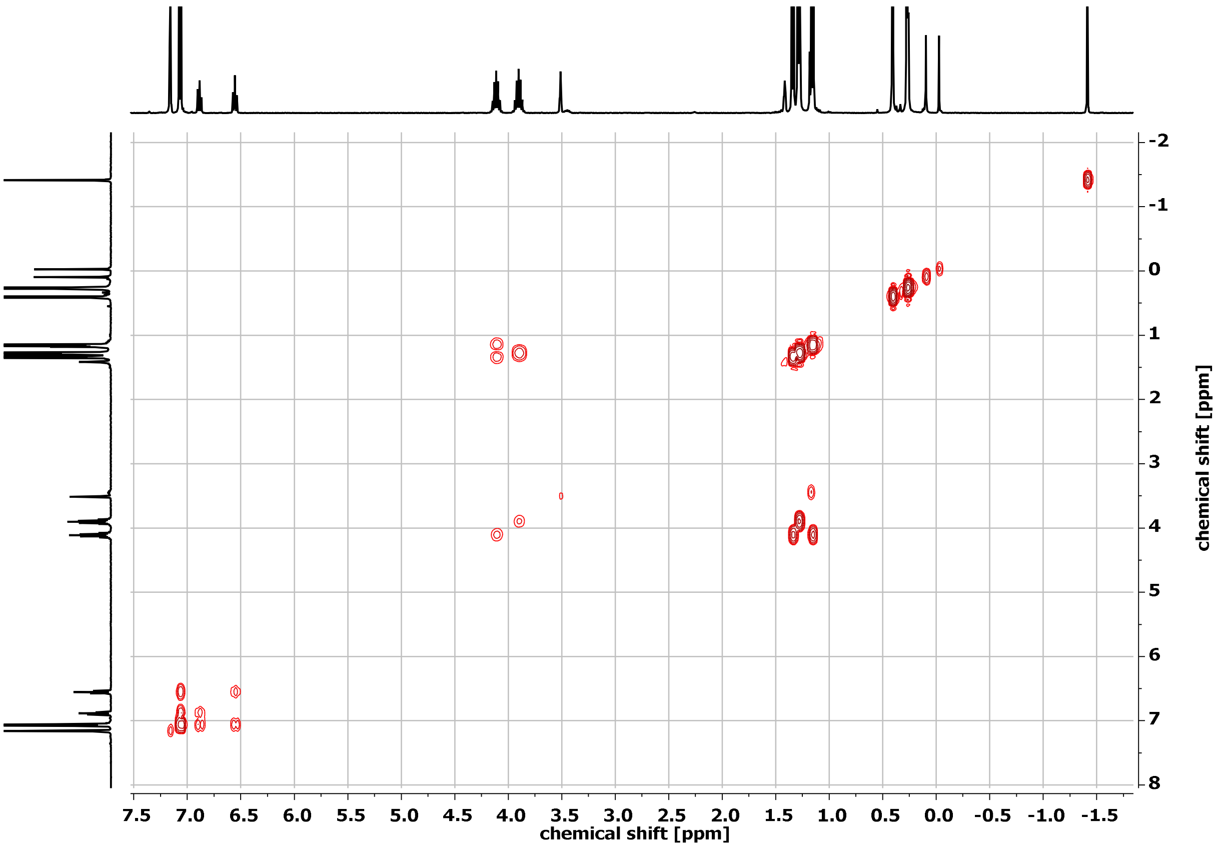

Figure S14 ^{1}H^{1}H-COSY NMR spectrum of $[(Cs)MgN'_2CH_2SiMe_3]_\infty$ **4** in a mixture of benzene-*d*$_6$:THF-*d*$_8$ (4:1).

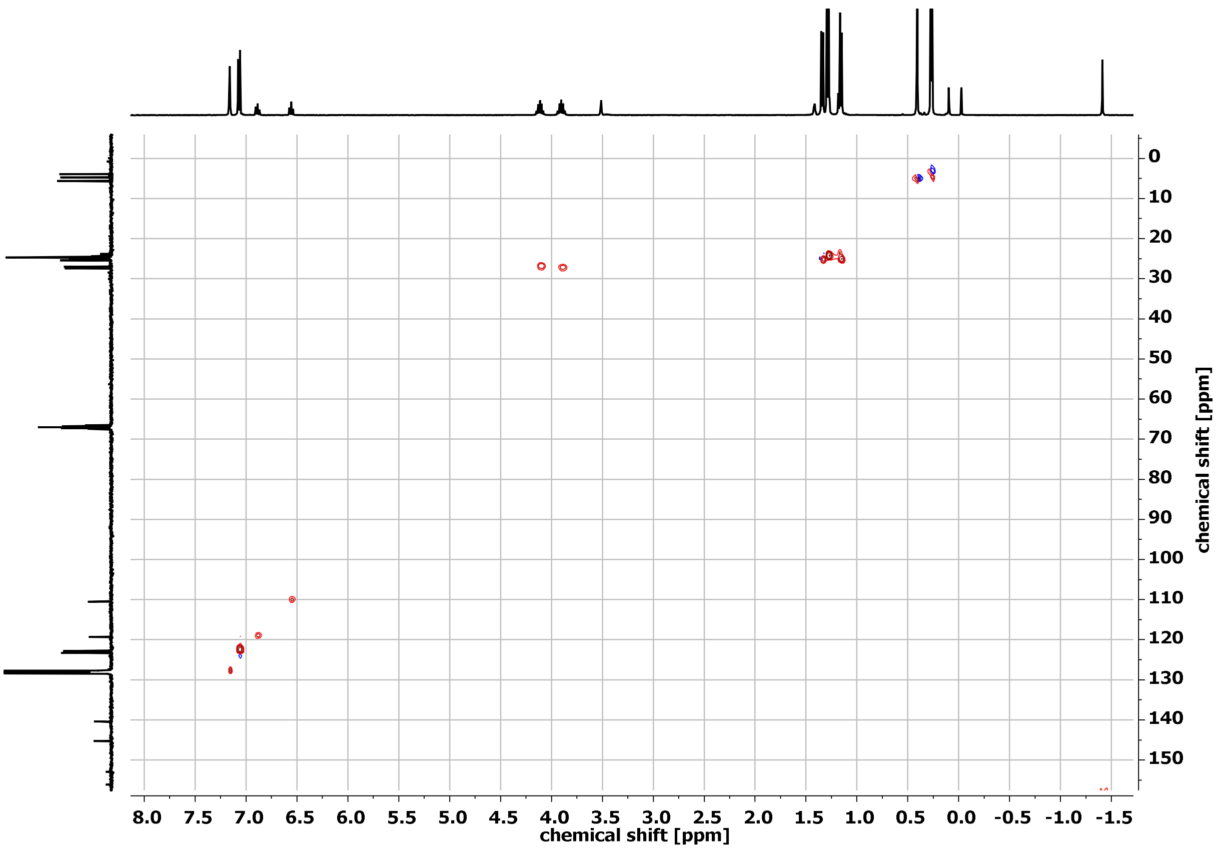

Figure S15 $^{1}H^{13}C$-HSQC NMR spectrum of $[(Cs)MgN'_2CH_2SiMe_3]_\infty$ **4** in a mixture of benzene-d_6:THF-d_8 (4:1).

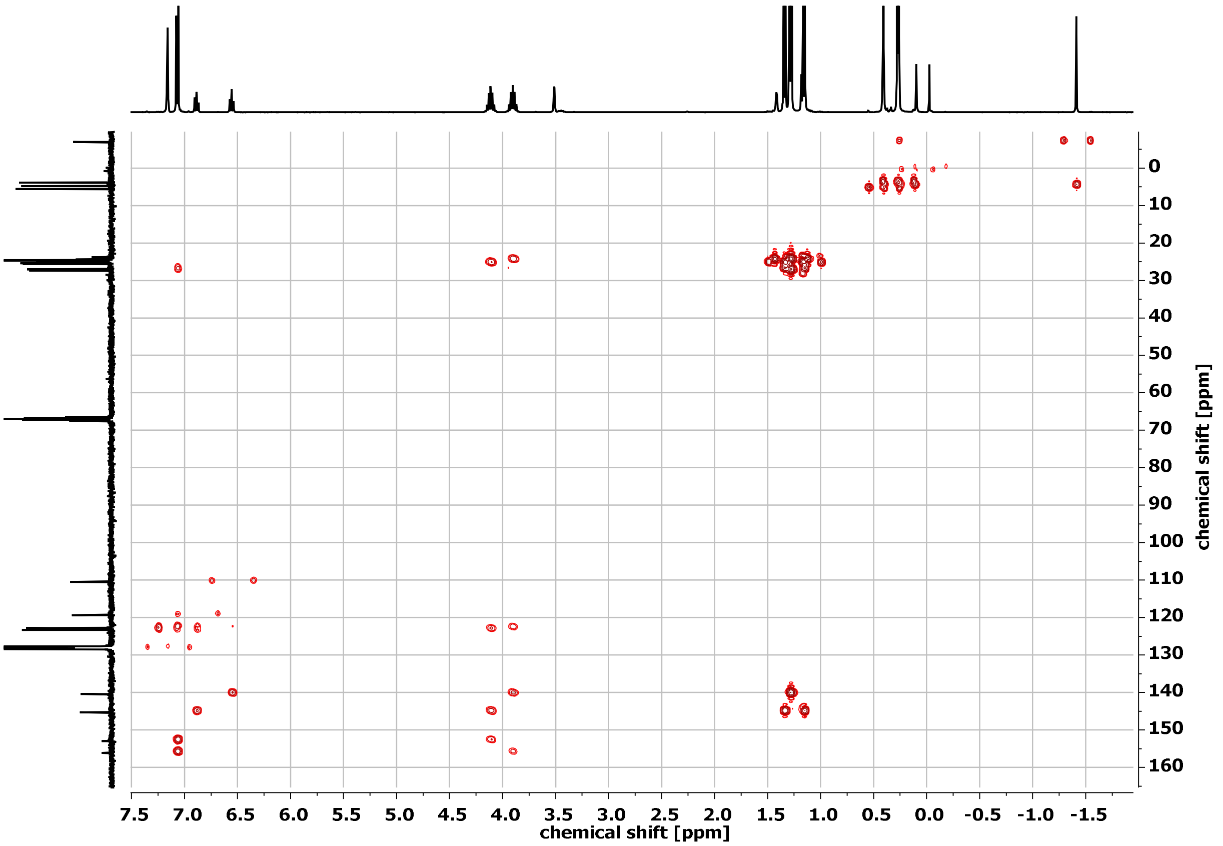

Figure S16 $^{1}H^{13}C$-HMBC NMR spectrum of $[(Cs)MgN'_2CH_2SiMe_3]_\infty$ **4** in a mixture of benzene-d_6:THF-d_8 (4:1).

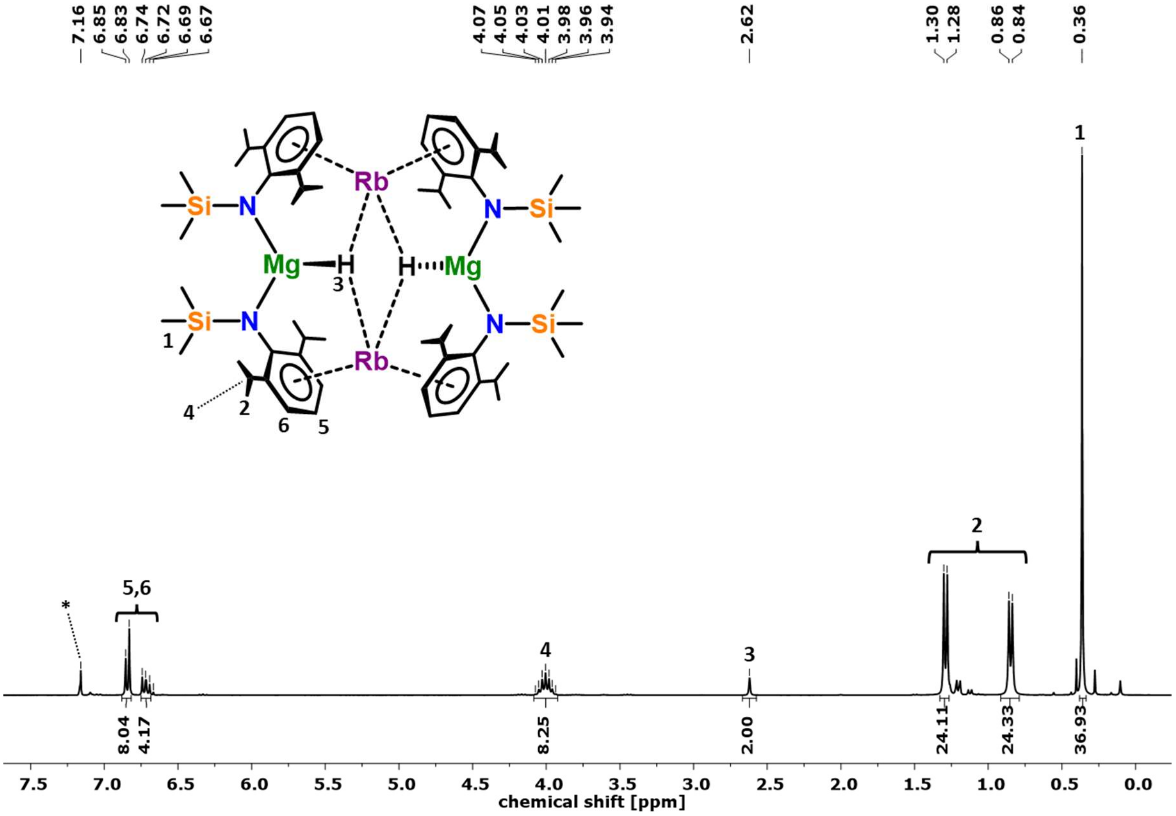

Figure S17 ^{1}H NMR spectrum of $[RbMgN'_2H]_2$ **5** in C_6D_6. Residual signal for deuterated solvent marked with *.

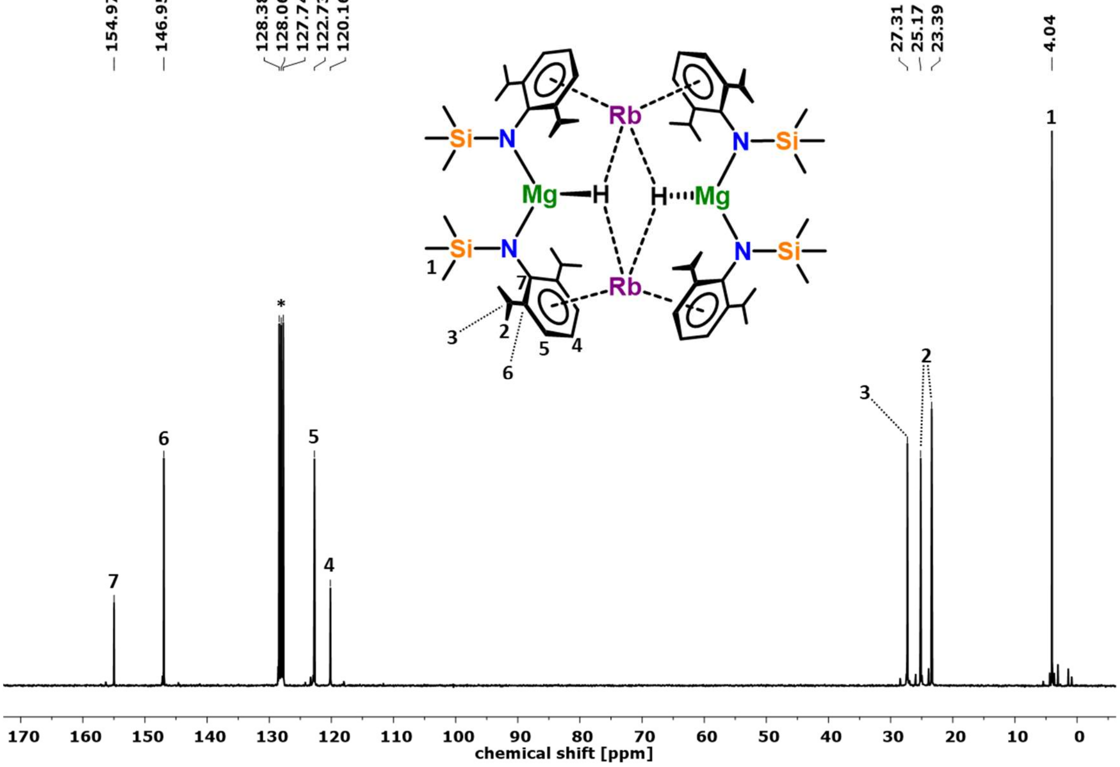

Figure S18 ^{13}C NMR spectrum of $[RbMgN'_2H]_2$ **5** in C_6D_6. Residual signal for deuterated solvent marked with *.

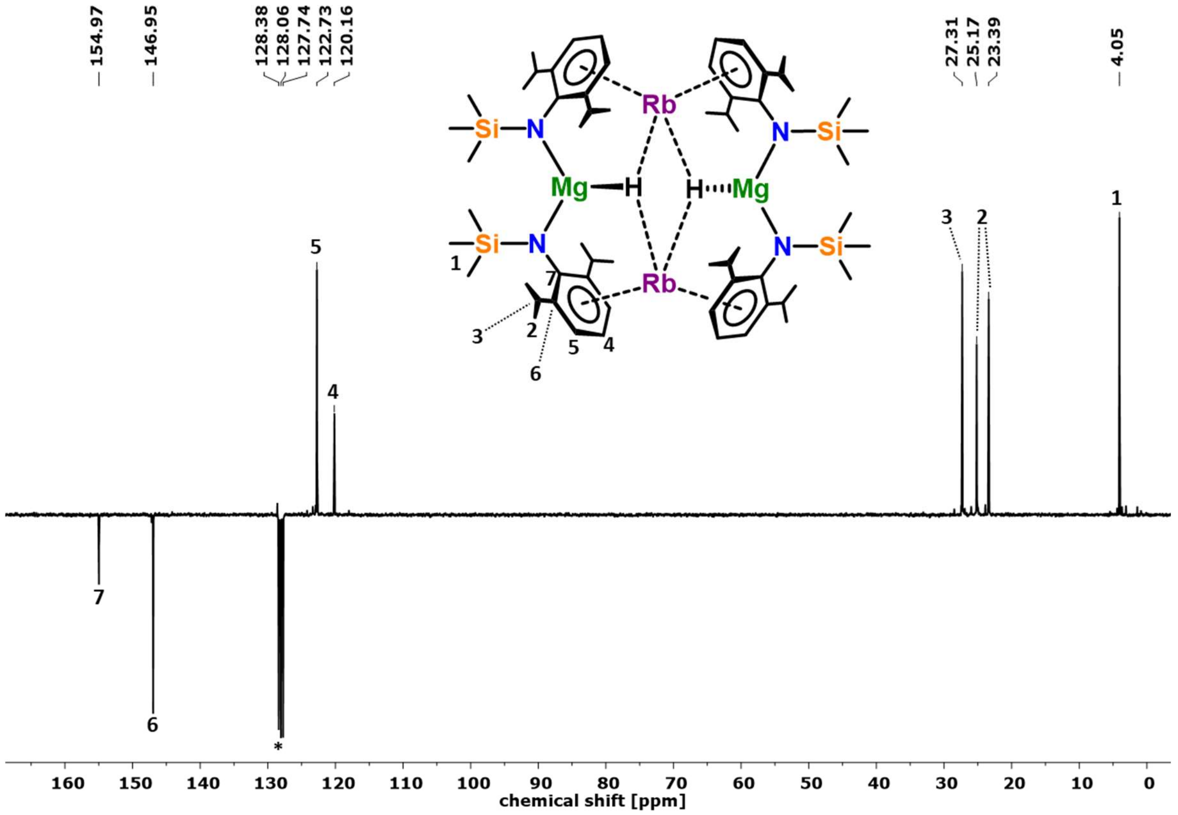

Figure S19 ^{13}C-DEPTQ NMR spectrum of $[RbMgN'_2H]_2$ **5** in C_6D_6. Residual signal for deuterated solvent marked with *.

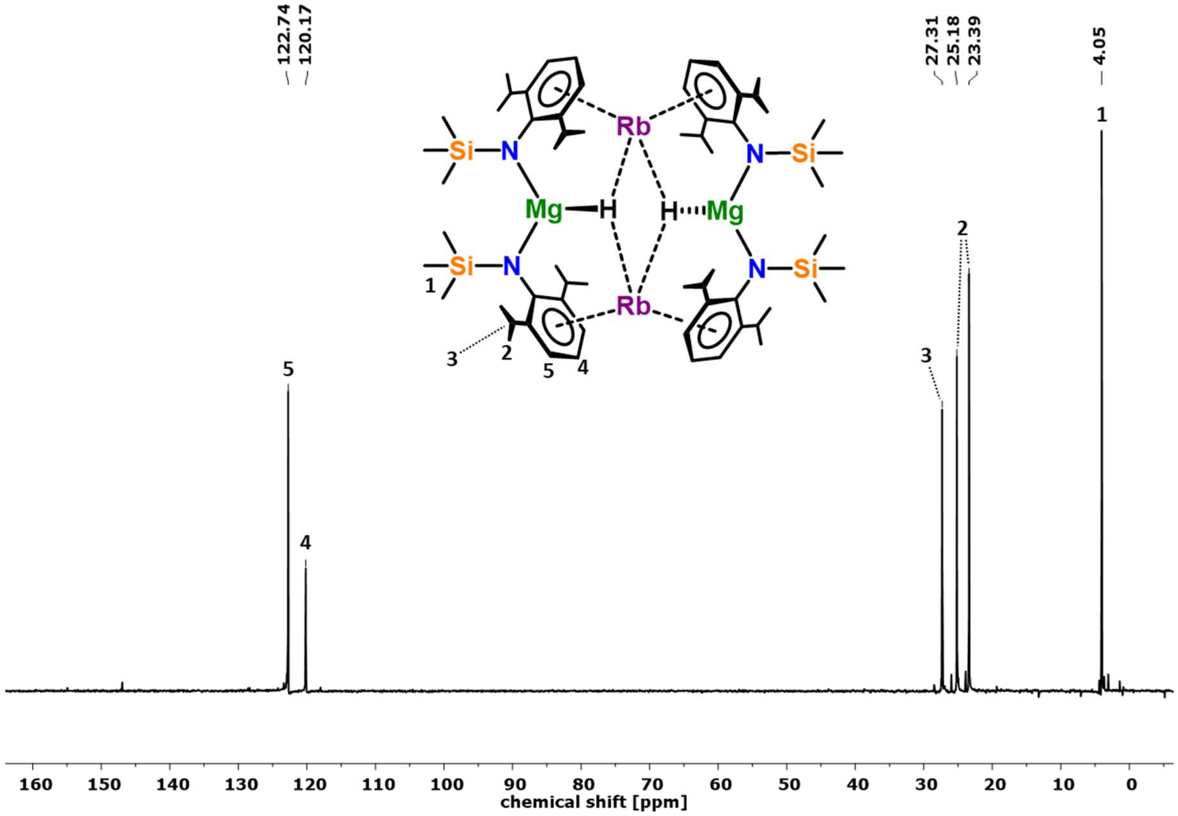

Figure S20 ^{13}C-DEPT-135 NMR spectrum of $[RbMgN'_2H]_2$ **5** in C_6D_6.

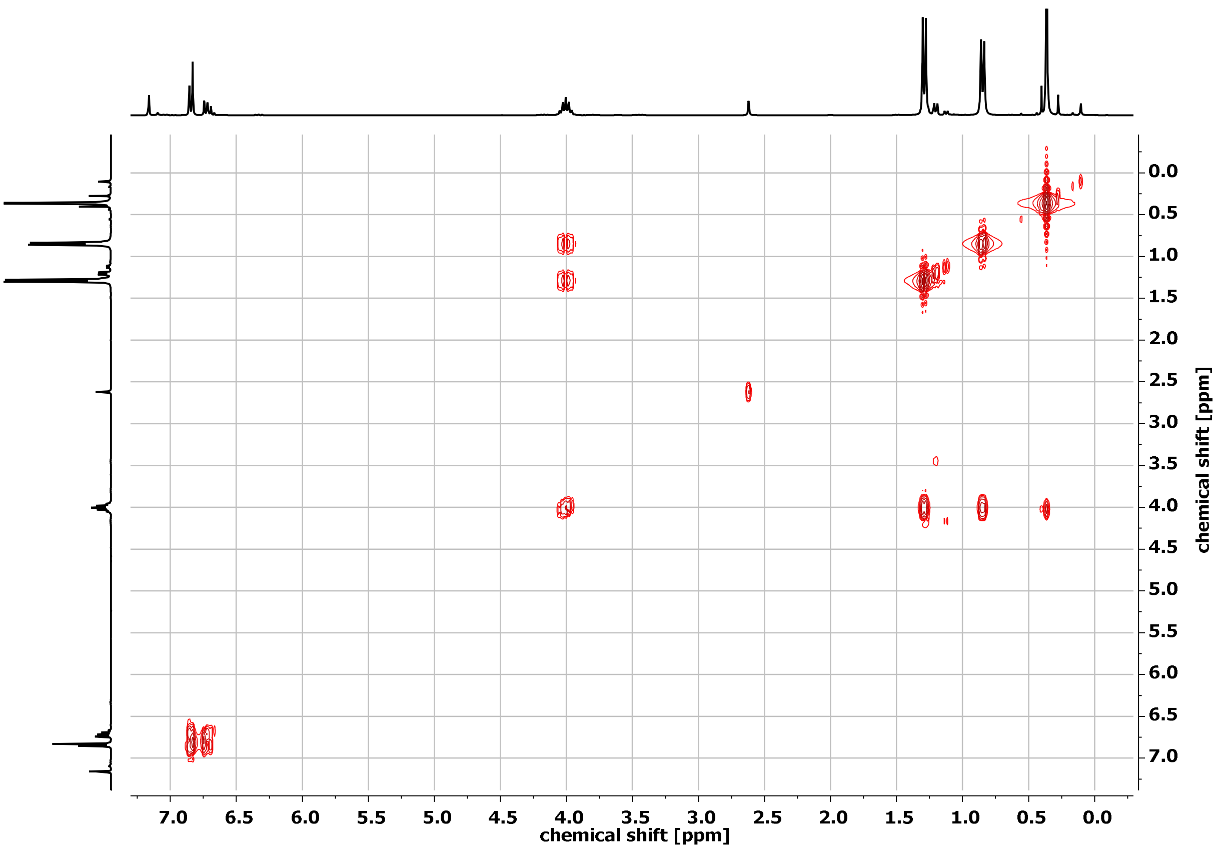

Figure S21 $^{1}H^{1}H$-COSY NMR spectrum of $[RbMgN'_{2}H]_{2}$ **5** in C_6D_6.

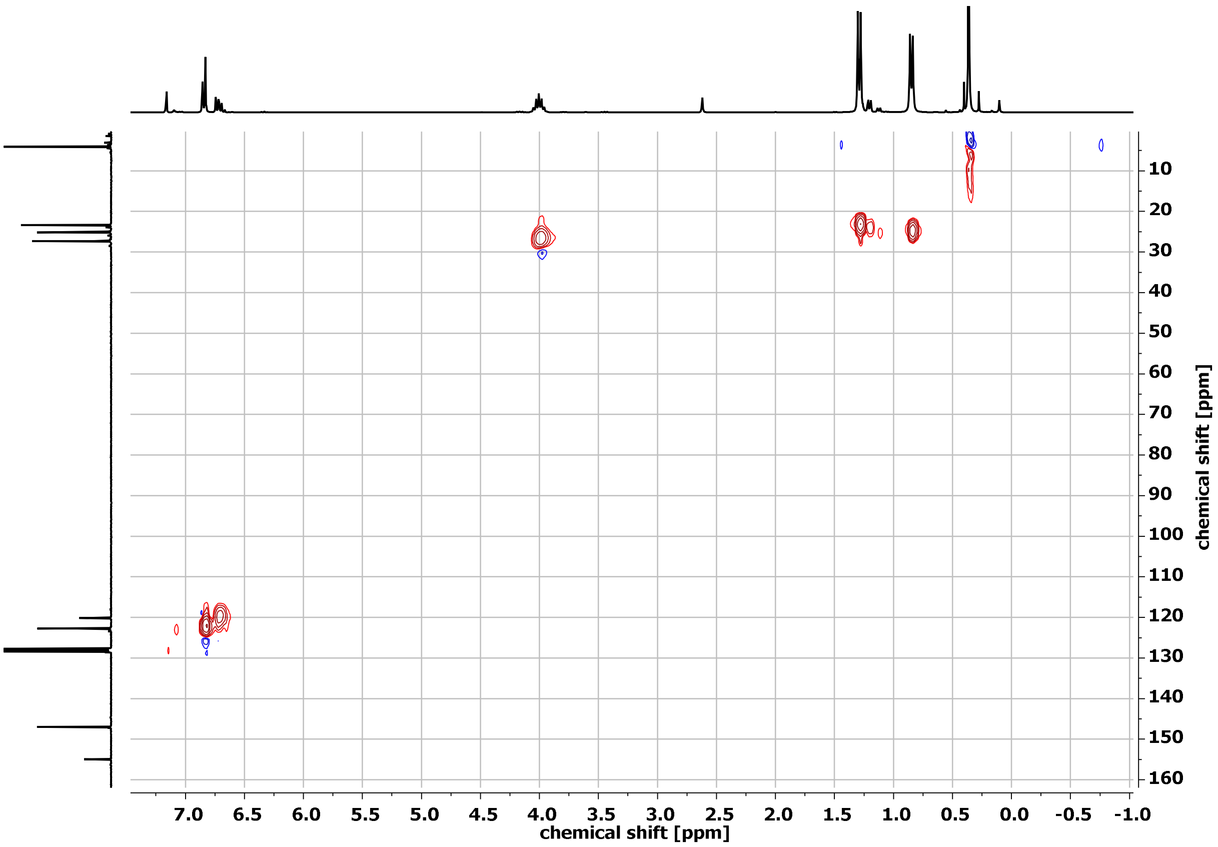

Figure S22 $^{1}H^{13}C$-HSQC NMR spectrum of $[RbMgN'_{2}H]_{2}$ **5** in C_6D_6.

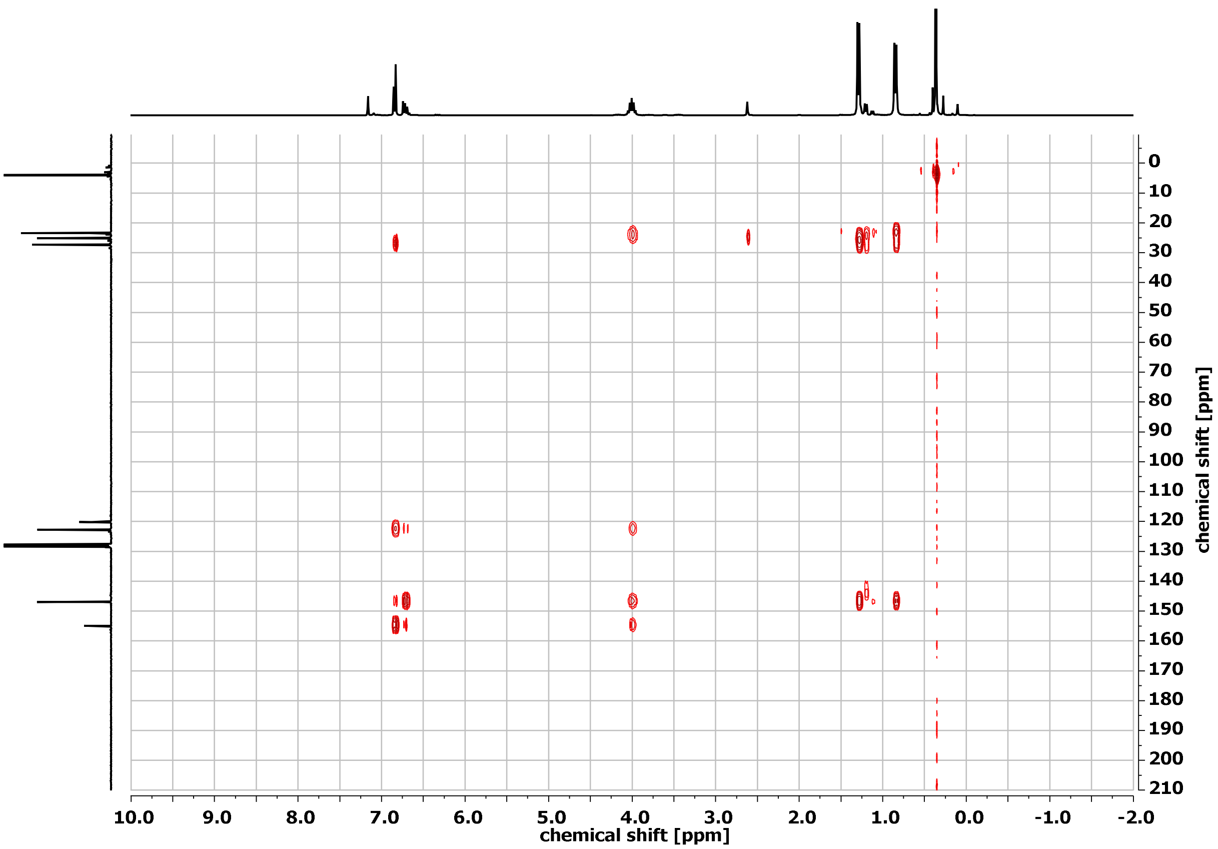

Figure S23 ^{1}H^{13}C-HMBC NMR spectrum of $[RbMgN'_2H]_2$ **5** in C_6D_6.
